# Carbonate anions and radicals induce interfacial water ordering in CO_2_ electroreduction on gold

**DOI:** 10.1038/s41557-025-01977-8

**Published:** 2025-11-25

**Authors:** Ya-Wei Zhou, Enric Ibáñez-Alé, Núria López, Beatriz Roldan Cuenya, Christopher S. Kley

**Affiliations:** 1 Helmholtz Young Investigator Group Nanoscale Operando CO2 Photo-Electrocatalysis, Helmholtz-Zentrum Berlin für Materialien und Energie GmbH, Berlin, Germany; 2 Department of Interface Science, Fritz Haber Institute of the Max Planck Society, Berlin, Germany; 3 Institute of Chemical Research of Catalonia (ICIQ-CERCA), The Barcelona Institute of Science and Technology (BIST), Tarragona, Spain

**Keywords:** Electrocatalysis, Catalytic mechanisms, Electrocatalysis

## Abstract

Interfacial hydration layers critically determine energy and chemical conversion processes, notably influencing the kinetics of electrocatalytic reactions. Fundamental mechanisms of reactions such as CO_2_ electroreduction and hydrogen evolution remain controversial due to the challenge of in situ deciphering of hydration structures alongside reaction intermediates and products. Here, by using vibrational and electrochemical spectroscopy paired with theory we reveal how carbonates structure interfacial water, affecting CO_2_ electroreduction and hydrogen evolution reactions on gold electrocatalysts in bicarbonate electrolytes. High cathodic potentials accelerate hydrogen evolution reactions by rapid proton delivery from ordered interfacial hydration networks, induced by carbonate molecules in equilibrium with their anion radicals. These radicals can serve, in addition to CO_2_, as a carbon source for CO and aldehyde production. Moreover we show water to be the primary proton donor for CO_2_ electroreduction and hydrogen evolution reactions, with bicarbonate mostly participating in the Heyrovsky step. Our molecular-level insights are relevant to rationalizing and optimizing electrochemical interfaces.

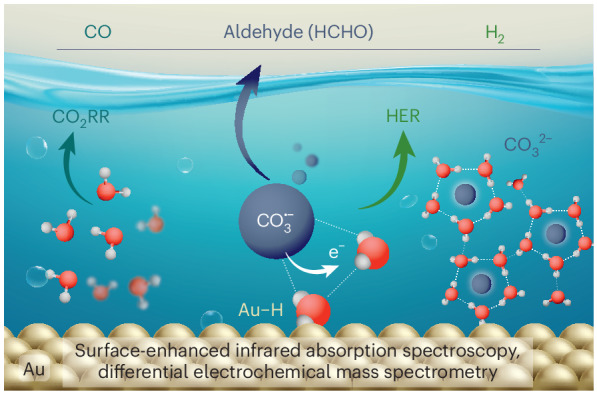

## Abstract (web summary)

The kinetics and product selectivity of electrocatalytic reactions depend on interfacial hydration, probing hydration structures alongside reaction intermediates and products is challenging. Now it has been shown that carbonates structure interfacial water during CO_2_ electroreduction and exist in equilibrium with their radicals, which serve various roles during the electrochemical processes.

## Main

Among the approaches employed towards closing the anthropogenic carbon cycle, the electrocatalytic CO_2_ reduction reaction (CO_2_RR) emerges as a promising route for converting atmospheric CO_2_ into valuable carbon fuels and chemicals^1–3^. However, its economic feasibility is compromised by low energy efficiency at high current densities, primarily attributed to the competing hydrogen evolution reaction (HER)^4^ and carbonate formation^5,6^. The kinetics of CO_2_RR depend on interfacial solvation, including the orientation of interfacial water and the hydrogen-bonding network within the electrical double layer (EDL) under an applied potential^7,8^. Thus, to understand the competitive relationship between CO_2_RR and HER, molecular-level insights on the nature of interfacial water within the EDL are essential. Previous studies have provided insights into the impact of hydration-water ordering on HER^9^, the hydrogen oxidation reaction^10,11^ or the oxygen reduction reaction^12^. These studies suggested that solvated cations promote a proton-coupled electron transfer reaction by influencing the interfacial water ordering in alkaline (MOH) electrolytes^13–15^. Cation effects have also been discussed in the context of facilitating CO_2_ adsorption or stabilizing ${\ast\atop}{\rm{C}}{{\rm{O}}}_{2}^{-}$ intermediates during CO_2_RR, but primarily in mildly acidic media, with little consideration given to the role of anionic carbonate species^16–18^. Despite extensive research in this field, the structure of interfacial water on metal electrodes during CO_2_RR has remained unclear, particularly due to the challenges of probing electrochemical interfaces by operando spectroscopies. It is crucial not only to resolve the interfacial hydration layer ordering but also to simultaneously identify the electrolyte and adsorbed electrode species (including transient reaction intermediates), both of which impact molecular ordering and, inherently, catalytic selectivity and reaction kinetics.

Elucidating the product formation rates and catalytic selectivities requires the identification of the proton donor and carbon source in CO_2_RR. Despite various species being suggested as the proton donor in CO_2_RR and HER, including water, hydronium, bicarbonate and carbonic acid, the major contributor remains debated^19–21^. In terms of carbon source, recent spectroscopic studies on gold (Au) electrodes in aqueous bicarbonate electrolytes have pointed to bicarbonate as the primary carbon source for CO formation. This was attributed to a rapid equilibrium between bicarbonate and CO_2_ (refs. ^22–30^), while diverging trends in potential-dependent CO adsorption on Au were reported^30,31^. Furthermore, previous studies primarily focused on linking the physical characteristics of metal electrode surfaces or the electrolyte microenvironment properties to catalytic performance^32–38^. For instance, Cheng et al. demonstrated that CO formation rates were suppressed by CO_2_ transport, which presents increasingly lower coverages at higher Au surface roughnesses^39^. Conversely, research has suggested that limited diffusion inhibits HER, thus maintaining high rates of CO_2_ conversion into CO on porous Au electrodes^21^. Koper et al. proposed that improved mass transport facilitates CO_2_RR by suppressing HER, attributed to the mitigation of local OH^−^ accumulation near the electrode^19^. Addressing the diverging interpretations regarding the competitive nature of HER and CO_2_RR on metal electrodes^40–42^ requires molecular-level investigations of the carbon source, proton donor and reaction intermediates, alongside their correlation with reaction kinetics and catalytic selectivity.

In this work we investigate the competing nature of CO_2_RR and HER on polycrystalline Au electrocatalysts in aqueous bicarbonate electrolyte. Using in situ attenuated total reflectance surface-enhanced infrared absorption spectroscopy (ATR-SEIRAS) and online differential electrochemical mass spectrometry (DEMS), we probe the interrelation between surface-adsorbed species, interfacial-water arrangement and reaction products. Leveraging Au as a well-established CO_2_ electrocatalyst, we provide insights into the proton donors and carbon sources of HER and CO_2_RR through isotope-labelling experiments and density functional theory (DFT). This integrated approach builds a comprehensive understanding of the dynamic electrochemical interface during CO_2_RR, guiding strategies to tune the selectivity and activity by controlling interfacial solution structures via electrolyte composition.

## Results

**Fig. 1:**
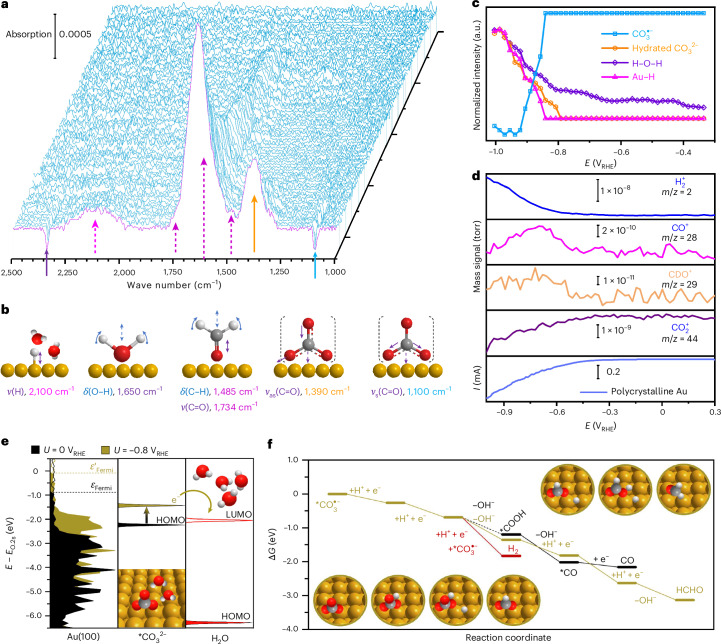
Real-time ATR-SEIRAS and DEMS spectra of CO_2_RR on polycrystalline Au and simulated carbonate anion radical reduction pathway. **a**,**b**, In situ p-polarized ATR-SEIRA spectra and schematics of observed vibrational fingerprints during CO_2_RR, respectively. **c**, Corresponding normalized IR band intensities. **d**, Online DEMS data and Faradaic current plot recorded on Au during CO_2_RR. Both operando ATR-SEIRAS and DEMS were measured in CO_2_-saturated 0.1 M KHCO_3_ with a scan rate of 2 mV s^−1^ and reference spectra recorded at 0.3 V_RHE_. **e**, Alignment of density of states, HOMOs and LUMOs of ${\ast\atop}{\rm{C}}{{\rm{O}}}_{3}^{2-}$ adsorbed on Au(100) and hydrated H_2_O (computed as a gas-phase H_2_O molecule surrounded by three explicit waters). *E* − *E*_O,2s_ refers to the energy of the density of states aligned to the energy of the 2s band centre of an O atom from the water molecule of reference. **f**, Gibbs energy (Δ*G*) profiles of ${{\rm{CO}}}_{3}^{\bullet -}$ reduction to formaldehyde (yellow) and CO (black), and competing HER (red), at *U* = –0.8 V_RHE_. Reaction intermediates are depicted with Au (yellow), C (grey), O (red) and H (white) atoms. *ν*, stretching mode; *ν*_s_, symmetric stretching; *ν*_as_, antisymmetric stretching; *δ*, scissoring mode; *E*, applied potential; *ε*_Fermi_, Fermi energy at *U* = 0.0 V; *ε*′_Fermi_, Fermi energy at *U* = −0.8 V; e^−^, electron. Source data

To identify the role of the different carbon and hydrogen sources, we ran individual experiments and correlated the most relevant fingerprints identified by infrared (IR) spectroscopy with DFT simulations. Scanning electron microscopy, energy dispersive X-ray spectroscopy and X-ray photoelectron spectroscopy data (Supplementary Figs. 1–4) confirm that the Au film fully covering the Si wafer remained intact during the ATR-SEIRAS measurements, with the observed IR signals originating from the Au surface. Consistently, the IR data recorded in 0.1 M KCl (Supplementary Fig. 5) show no signals from the Si substrate, further supporting cross-sectional scanning electron microscopy, energy dispersive X-ray spectroscopy mapping and surface X-ray photoelectron spectroscopy results. Figure 1a depicts the ATR-SEIRAS spectra obtained on polycrystalline Au in CO_2_-saturated 0.1 M KHCO_3_ electrolyte during a potential sweep from +0.3 to −1.0 V versus reversible hydrogen electrode (RHE; V_RHE_). Figure 1b,c shows the surface species corresponding to the observed vibrational fingerprints and the potential-dependent normalized band intensity profiles, respectively. The assignments of all IR vibrational bonds are summarized in Supplementary Table 1. The IR peak at ~2,340 cm^−1^ is attributed to dissolved CO_2_, which diminishes as CO_2_ is consumed with increasing cathodic potential. The band at ~1,390 cm^−1^ is assigned to adsorbed carbonate $\left({{\rm{CO}}}_{3}^{2-}\right)$, consistent with previously reported spectral fingerprints of carbonate ions in 0.1 M Na_2_CO_3_ (ref. ^43^). At increasing cathodic potentials, more OH^−^ is formed through HER and carbonate ions accumulate on the Au surface, as evidenced by the increasing intensity of the carbonate band (Fig. 1c). Conversely, in Ar-saturated electrolyte (Supplementary Fig. 6b), the intensity of the carbonate band decreases at potentials below −0.75 V_RHE_, indicating carbonate consumption.

Notably, an IR peak at ~1,100 cm^−1^ is observed (Fig. 1a). To assign this fingerprint, we computed the frequencies of carbonate $\left({{\rm{CO}}}_{3}^{2-}\right)$ and carbonate anion radical $\left({{\rm{CO}}}_{3}^{\bullet -}\right)$ bidentate adsorption (*η*_O,O_) on (100) and (111) Au surfaces (DFT structural models in Supplementary Fig. 9). The assignment of ${{\rm{CO}}}_{3}^{\bullet -}$ is corroborated by ^13^C-labelled IR measurements (Supplementary Fig. 13b), which show no detectable isotope shift in the symmetric stretching mode of the ${{\rm{CO}}}_{3}^{\bullet -}$ vibrational mode (*ν*_s_(C–O)) due to its symmetric bonding configuration, with the stretching mode being less sensitive to isotope substitution. By contrast, the asymmetric C–O stretching mode of ${{\rm{CO}}}_{3}^{2-}$ exhibits a pronounced shift from 1,390 cm^−1^ to 1,352 cm^−1^, as expected for out-of-phase oxygen motion (Supplementary Fig. 13c). While the obtained values for unsolvated ${v}_{3}({\mathrm{CO}}_{3}^{2-})$ were close to the ~1,390 cm^−1^ band, ${v}_{1}$ shifted from 1,201–1,202 cm^−1^ to 1,091–1,093 cm^−1^ when the corresponding anion radical carbonate was computed (Fig. 1b and Supplementary Fig. 7). The shift in the band is due to the anisotropic nature of the radical that has two short C–O bonds and one long bond, and the radical character is localized in the most external O atom. Explicit solvation via *n*H_2_O molecules (*n* = 1–3, considering that two explicit water molecules are enough to properly compute the hightest occupied molecular orbital (HOMO) energy levels^44^) revealed that the *ν*_1_ mode shifted even closer to ~1,100 cm^−1^ for *n* = 1 and 2 on the (100) surface (Supplementary Fig. 10), while the frequencies on the (111) surface remained unaffected. Hence, the simulated ${{\rm{CO}}}_{3}^{\bullet -}$ on Au(100) with two H_2_O molecules best represents the species appearing at ~1,100 cm^−1^ in the IR results. Another important factor is that the presence of carbonate (or its anion radical) strongly pins the water structure around it. The H_2_O molecules around the ${{\rm{CO}}}_{3}^{\bullet -}$ configuration in Fig. 1e are more stable compared with liquid water (by around 0.2 eV). Nevertheless, ${{\rm{CO}}}_{3}^{2-}$ adsorption energies are not favoured even when including up to three explicit H_2_O molecules; thus, the computational hydrogen electrode (CHE) approach was benchmarked with grand canonical DFT to include the potential and also the implicit solvent and cations (see ‘Computational Details’ in Methods). A stabilization trend for ${{\rm{CO}}}_{3}^{2-}$ is observed as the applied potential by grand canonical DFT becomes more cathodic (Supplementary Fig. 7), in contrast with the destabilization trend found for CHE, and when two explicit H_2_O molecules are included, the adsorption energy becomes exergonic at potentials more negative than –0.8 V_RHE_, in good agreement with its experimental detection window (Fig. 1c).

The ${{\rm{CO}}}_{3}^{\bullet -}$ band was also observed in the Ar-saturated electrolyte (Supplementary Fig. 6a). The similar band intensity and slightly shifted maximum peak position with respect to the IR band of carbonate (Supplementary Fig. 6b) indicate that the formation of the carbonate radical ion $\left({{\rm{CO}}}_{3}^{\bullet -}\right)$ is linked to carbonate. Upon the addition of KHCO_3_ into Ar-saturated H_2_O, the ${{\rm{CO}}}_{3}^{\bullet -}$ band appears within the first several minutes, demonstrating the conversion of ${{\rm{CO}}}_{3}^{2-}$ into ${{\rm{CO}}}_{3}^{\bullet -}$. Overall, carbonate and the carbonate anion radical appear concurrently, and ${{\rm{CO}}}_{3}^{\bullet -}$ is formed via charge transfer from carbonate to the water hydration shell. This charge transfer is more likely to happen when a cathodic potential is applied, as the ${{\rm{CO}}}_{3}^{2-}$ HOMO is shifted to higher energy levels than the hydrated H_2_O molecule’s lowest unoccupied molecular orbital (LUMO), thus enabling electron transfer (Fig. 1e). We also assessed this band alignment for carbonate adsorbed on (100) slabs of other metals (Ag, Cu, Pd and Pt), and the unique proximity of the ${\rm{Au}}(100)-{\ast\atop}{\rm{C}}{{\rm{O}}}_{3}^{2-}$ HOMO energy level to the LUMO of hydrated H_2_O indicates the capacity of Au surfaces to mediate this charge transfer to water-generating carbonate radicals (Supplementary Fig. 11). Furthermore, the increasingly negative ${{\rm{CO}}}_{3}^{\bullet -}$ band (Fig. 1a,c) suggests that carbonate anion radicals are reduced and potentially serve as additional carbon sources.

In addition, experimentally, IR bands at ~1,734 cm^−1^ and ~1,500 cm^−1^ are observed, aligning with the vibrational frequencies of C=O and the CH_2_ bonds of formaldehyde. Complementary high-sensitivity DEMS measurements (Fig. 1d) provide molecular-level evidence for the formation of formaldehyde species (CHO^+^, mass to charge ratio of *m*/*z* = 29) during CO_2_RR on Au. A similar band at ~1,720 cm^−1^ has been observed on Cu electrodes and assigned to adsorbed formyl (*CHO)^28,45^. A minor amount of formaldehyde is also detected in Ar-saturated electrolyte, as evidenced by the enhanced C=O and CH_2_ band intensities (Supplementary Fig. 6) and the detected mass signal of CHO^+^ (Supplementary Fig. 8). This observation suggests the involvement of a reactant besides CO_2_ in CO_2_RR, as discussed further below.

Previous studies have shown that the reactant for CO_2_RR may not be solely dissolved CO_2_, but could also include CO_2_ derived from an equilibrium reaction with ${{\rm{HCO}}}_{3}^{-}$ (refs. ^28,29^). As shown in Supplementary Fig. 16a,b, we observe the simultaneous presence of ${{\rm{HCO}}}_{3}^{-}$, CO_2_ and ${{\rm{CO}}}_{3}^{2-}$ bands, confirming the acid–base equilibria between ${{\rm{HCO}}}_{3}^{-}$ and CO_2_ (2${{\rm{HCO}}}_{3}^{-}$ ↔ CO_2_ + H_2_O + ${{\rm{CO}}}_{3}^{2-}$). Quantitative DEMS analysis indicates that the Au electrode does not encounter CO_2_ mass-transfer limitations (Supplementary Fig. 15) and that ${{\rm{HCO}}}_{3}^{-}$ is not the primary carbon source (Supplementary Fig. 16). In the Ar-saturated electrolyte, the presence of CO and HCHO is observed (Supplementary Fig. 8), with the CO_2_ originating entirely from the rapid equilibrium between ${\mathrm{HCO}}_{3}^{-}$ and CO_2_. However, compared with the CO_2_-saturated electrolyte, less than one-tenth of the consumed CO_2_ yields approximately half the quantity of CO and HCHO in the Ar-saturated electrolyte, suggesting that dissolved CO_2_ is not the unique carbon source. The in situ ATR-SEIRAS and online DEMS data indicate that the carbonate radical acts as an additional carbon source during CO_2_RR, undergoing reduction into HCHO and CO on Au electrodes.

To further rationalize these observations, we computed the energy profiles of CO and HCHO on Au(100), considering both ${{\rm{CO}}}_{3}^{\bullet -}$ and CO_2_ as precursors. In Fig. 1f, an initial hydrogenation of carbonate anion radical to *HCO_3_ followed by a co-adsorption of *H is considered, which can proceed with two consecutive *H nucleophilic attacks, the first generating *HCO_2_ and OH^−^, and the second producing HCHO and OH^−^. Alternatively, *HCO_3_ and co-adsorbed *H can evolve to OH^−^ and *COOH, the last being the common intermediate leading to CO^2^, or the *H could instead undergo a Heyrovsky step to produce H_2_ (ref. ^46^). Both ${{\rm{CO}}}_{3}^{\bullet -}$ reduction pathways to HCHO (yellow) and CO (black) show downhill energy profiles at *U* = −0.8 V_RHE_, where *U* is the applied potential by means of computational hydrogen electrode, with formaldehyde being more favoured, as the ${\ast\atop}{\rm{H}}{{\rm{CO}}}_{2}$ intermediate is more stable than *COOH (Fig. 1e). However, HER dominates over both pathways, as the co-adsorbed *H would rather evolve to H_2_ (red), in agreement with the increasing ${{\rm{H}}}_{2}^{+}$ detection by DEMS at high cathodic potentials (Fig. 1d). Moreover, we also investigated the CO_2_ reduction to HCHO compared with the well-known pathway to CO on Au (refs. ^19,47^), considering that the *CO intermediate can alternatively be hydrogenated to HCHO instead of desorbing. Both profiles at *U* = –0.6 V_RHE_ show endergonic character for the rate-determining steps (0.05 and 0.32 eV for *CO desorption and hydrogenation to ${\ast\atop}{\rm{H}}{\rm{CO}}$, respectively), but at more cathodic potentials, the profile of CO_2_RR to CO becomes downhill after the ${\ast\atop}{\rm{C}}{{\rm{O}}}_{2}^{-}$ adsorption step, and is thus more favoured than HCHO generation (Supplementary Fig. 17). These computed profiles indicate that both CO_2_ and ${{\rm{CO}}}_{3}^{\bullet -}$ act as a carbon source for HCHO and CO, suggesting that the two CHO^+^ DEMS signals obtained at –0.6 V_RHE_ and –0.9 V_RHE_ (Fig. 1d) can be assigned to HCHO produced from CO_2_ and ${{\rm{CO}}}_{3}^{\bullet -}$, respectively.

At ~2,100 cm^−1^, an IR band is detected below –0.84 V_RHE_ in CO_2_-saturated electrolyte (Fig. 1a) and between –0.56 and –0.77 V_RHE_ in Ar-saturated electrolyte (Supplementary Fig. 6a). This band originates from an adsorbed hydrogen atom bonded to the gold surface (Au–H), consistent with previous findings based on in situ surface-enhanced Raman spectroscopy^13^. The assignment is further supported by the appearance of Au–D vibrational bands in the D_2_O labelling experiments (Supplementary Fig. 12), while the absence of a 2,100 cm^−1^ feature in D_2_O electrolyte confirms that no CO-related vibrations arise from the Au surface under cathodic polarization. The broadened Au–H band indicates variations in coverage, bonding configuration or interactions with interfacial water^48–51^. The overlap between the O–D bending mode and the Au–D band (Supplementary Fig. 12b) and the transition from adsorbed hydrogen to interfacial water (Supplementary Fig. 13b) demonstrate a coexistence and competitive balance on the Au electrode under reducing conditions. Moreover, the IR peak at ~1,650 cm^−1^ is attributed to the bending mode *δ*(H–O–H) of adsorbed water molecules (Fig. 1a and Supplementary Fig. 6a). In both CO_2_-saturated electrolytes (Fig. 1c) and Ar-saturated electrolytes (Supplementary Fig. 6c), the intensity of the Au–H band (*H) shows a linear correlation with the *δ*(H–O–H) bending mode, indicating a link between *H formation and the decomposition of interfacial water. A similar trend is observed in the IR band intensity of interfacial H_2_O and ${{\rm{CO}}}_{3}^{2-}$ (Fig. 1c and Supplementary Fig. 6c), which indicates that the presence of carbonates induces structuring of interfacial water molecules. Notably, there is both experimental and theoretical evidence for the formation of stable hydrated ${{\rm{CO}}}_{3}^{2-}$ molecular clusters in the liquid phase^43,52^.

**Fig. 2:**
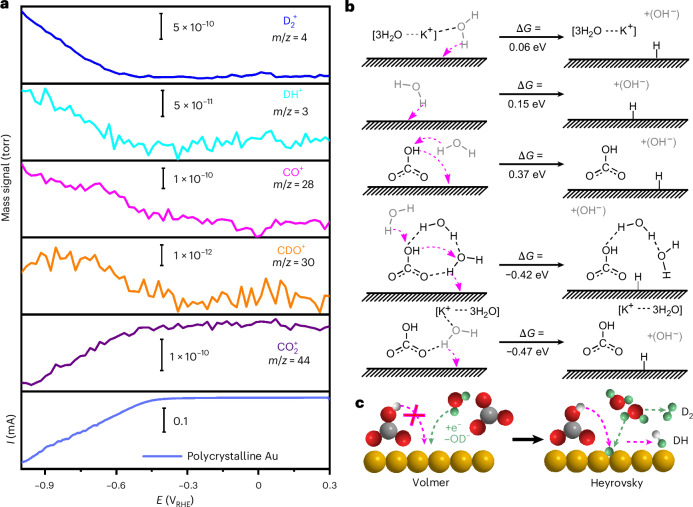
Identifying the proton donor of HER and CO_2_RR. **a**, D_2_O isotopic labelling in online DEMS and the faradaic current plot recorded on a polycrystalline Au electrode in CO_2_-saturated 0.1 M KHCO_3_ with a scan rate at 2 mV s^−1^. **b**, Formation energies of Au–H in reactions from free H_2_O, ${{\rm{HCO}}}_{3}^{-}$ and hydrated carbonate on the (100) surface at a potential of –0.8 V_RHE_. **c**, Schematic Volmer and Heyrovsky steps on the Au surface. Depicted atoms are Au (yellow), C (grey), O (red), H (white) and D (green). Source data

To ascertain the role of interfacial hydration water as a proton donor in HER and CO_2_RR and to elucidate the competing mechanisms of the latter, we conducted isotopic D_2_O labelling DEMS measurements on Au in CO_2_-saturated 0.1 M KHCO_3_. Figure 2a depicts the cathodic current alongside the corresponding mass signals of detected products and CO_2_. In the CO_2_-saturated D_2_O electrolyte, mass signals corresponding to D_2_ (*m*/*z* = 4) and DH (*m*/*z* = 3) were detected, indicating that both water (D_2_O) and bicarbonate serve as proton donors for HER. Additionally, alongside D_2_, CO and DH, the mass signal of CDO (*m*/*z* = 30) was detected during negative potential sweeps, suggesting that *D derived from water (D_2_O) acts as the proton donor of CO_2_RR. That the formation of adsorbed hydrogen (the Volmer step) is the first and rate-determining step of HER on Au in both acidic and alkaline media is widely accepted^13^. This step is followed either by the Heyrovsky or Tafel step: (1) a recombination of the adsorbed hydride (*H^−*δ*^) and another solvated proton to generate molecular hydrogen (Heyrovsky step); or (2) coupling of two adsorbed hydrogen atoms to form hydrogen (Tafel step). However, the Tafel step is unlikely to occur on Au in KHCO_3_ electrolyte due to weak H adsorption and consequently low coverage in neutral or basic media. The observation of D_2_ and DH and the absence of a mass signal for H_2_ (*m*/*z* = 2) indicates that water molecules participate in both the Volmer and Heyrovsky steps, while ${{\rm{HCO}}}_{3}^{-}$ participates only in the Heyrovsky reaction.

To validate the identification of the proton donor, we computed the thermodynamics of the Volmer step on the Au surface considering contributions from explicit hydrated K^+^, free water, bicarbonate and the hydration layer of carbonate, as illustrated in Fig. 2b. The Gibbs free energies associated with H^+^ delivery to the surface follow this order: ${{\rm{CO}}}_{3}^{2-}$ hydration layer (−0.42 eV) < hydrated K^+^ (0.06 eV) < free water (0.15 eV) < bicarbonate (0.37 eV). This suggests that the Volmer step via the decomposition of interfacial water in the presence of hydrated carbonate is the most plausible mechanism for the enhancement of HER at cathodic potentials. Moreover, adding the cation to the existing carbonate-mediated mechanism decreases the energy only from –0.42 eV to –0.47 eV, indicating the dominant effect of carbonates on enhancing proton migration to the surface. Therefore, the HER enhancement effects ascribed to cations in the literature must correlate with the kinetics rather than the carbonates’ favoured thermodynamics. We also assessed the effect of explicit K^+^ on ${\ast\atop}{\rm{C}}{{\rm{O}}}_{3}^{2-}$ stability on Au(100), which shows a decrease on its adsorption free energy (∆∆*G*_ads_ = –1.30 eV), thus indicating that cations also play an indirect role on HER by stabilizing the carbonates that enhance the water splitting and Au–H formation. The identification of water as the proton donor for both HER and CO_2_RR is corroborated by the in situ IR spectra, indicating that the formation of *H depends on the decomposition of interfacial H_2_O. Moreover, the approximately tenfold higher mass signal intensity of D_2_ compared with DH implies that the interfacial water acts as the predominant proton source in the Heyrovsky step as well. Consequently, the HER mechanism on Au can be summarized in the elementary steps illustrated in Fig. 2c.

**Fig. 3:**
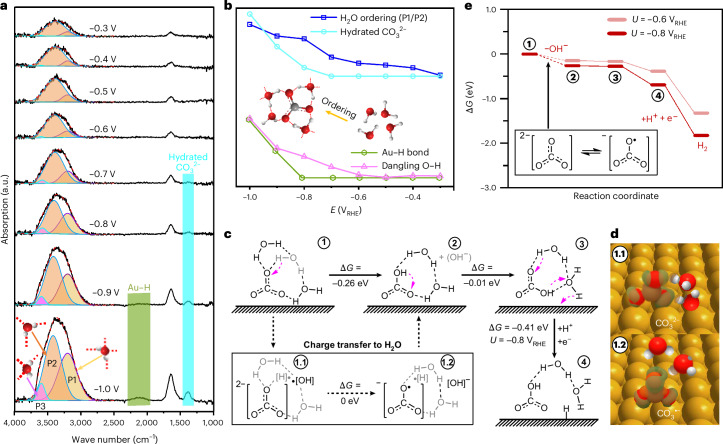
In situ ATR-SEIRAS spectra of interfacial H_2_O and simulated HER pathway from hydrated ${{\rm{CO}}}_{3}^{2-}$ water layer. **a**,**b**, In situ ATR-SEIRA spectra of interfacial H_2_O (**a**) and corresponding normalized band intensity (**b**) recorded on a polycrystalline Au electrode in CO_2_-saturated 0.1 M KHCO_3_. Gaussian fits of three O–H stretching modes shown as yellow (peak 1, P1), orange (peak 2, P2) and purple (peak 3, P3). **c**, Reaction pathway of Au–H formation from interfacial H_2_O layer on Au(100). **d**, Charge density differences due to charge transfer from ${{\rm{CO}}}_{3}^{2-}$ and ${{\rm{CO}}}_{3}^{\bullet -}$ to interfacial H_2_O. In the panels Au, C, O and H atoms are depicted in yellow, grey, red and white, respectively; depletion and accumulation of charge density are depicted in blue and orange, respectively. **e**, Gibbs energy profiles of HER enhanced by ${{\rm{CO}}}_{3}^{\bullet -}$. Reference spectrum recorded at 0.3 V_RHE_. Source data

To gain mechanistic insights into the competitive interplay between CO_2_RR and HER, we investigate the structure of interfacial water within the EDL on Au under CO_2_RR conditions. As shown in Fig. 3a and Supplementary Fig. 19a, the potential-dependent intensity of the stretching H–O–H band exhibits a linear correlation with the presence of hydrated ${{\rm{CO}}}_{3}^{2-}$ in both Ar-saturated and CO_2_-saturated electrolytes. This observation suggests that hydrated ${{\rm{CO}}}_{3}^{2-}$ influences the structure of the interfacial water network on Au surfaces. Moreover, the broad H_2_O band shifts towards lower wave numbers with the increasing intensity of hydrated ${{\rm{CO}}}_{3}^{2-}$ (Supplementary Fig. 18), indicating charge transfer from carbonate to hydration water and the formation of shorter hydrogen bonds facilitated by ${{\rm{CO}}}_{3}^{2-}$. This O–H stretching band, observed both in CO_2_-saturated (Fig. 3a) and Ar-saturated (Supplementary Fig. 19a) electrolytes, was deconvoluted into three distinct peaks via Gaussian fitting. The low-frequency peak at ~3,200 cm^−1^ (peak 1) is attributed to the tetrahedral structure of water featuring tetrahedral-coordinated hydrogen bonds, resembling the well-ordered structure of ice. The band at ~3,400 cm^−1^ (peak 2) is assigned to water molecules with trihedral-coordinated hydrogen bonds, representing disordered liquid water. The peak at ~3,600 cm^−1^ (peak 3) corresponds to water molecules featuring dangling O–H bonds, lacking hydrogen-bond interactions with neighbouring water molecules. The intensity ratio of peak 1 and peak 2 serves as a measure of the degree of interfacial water structure ordering. Additionally, the O–H stretch shifts are plotted in Supplementary Fig. 18 to further illustrate the evolution of water ordering under applied bias.

As shown in Fig. 3b, the ordering of interfacial water correlates with the increasing intensity of hydrated ${{\rm{CO}}}_{3}^{2-}$ in CO_2_-saturated electrolyte, indicating that carbonate species facilitate the formation of an ordered interfacial water layer. Similarly, in Ar-saturated electrolyte (Supplementary Fig. 19b), a comparable linear relationship between the interfacial water ordering and the intensity of the hydrated ${{\rm{CO}}}_{3}^{\bullet -}$ band is observed, underscoring the promoting effect of carbonates in fostering well-ordered hydration networks. Previous spectroscopic studies have suggested an interfacial-water-molecule transition during HER, in the presence of cations, from a disordered arrangement to an ordered structure^7,10^. This cation effect was further confirmed by the increased intensity of the interfacial H_2_O bands recorded in 0.1 M KCl electrolyte (Supplementary Fig. 5), indicating the formation of an ordered interfacial water layer as solvated cations accumulate on Au at more negative potentials. Here the promotion of the ordered interfacial hydration layers is rationalized by the additional influence of carbonate ions in the bicarbonate electrolyte. In Ar-saturated electrolyte, the ordering of the interfacial water (Supplementary Fig. 19) initially increases with a negative potential sweep but decreases below –0.7 V_RHE_, correlating directly with the intensity of the hydrated ${{\rm{CO}}}_{3}^{2-}$ IR bands rather than with the cation concentration, which is expected to increase at these cathodic potentials. These spectroscopic data demonstrate that the specific adsorption of ${{\rm{CO}}}_{3}^{2-}$ ions with adjacent hydration water molecules via hydrogen bonds (Supplementary Fig. 18) forms an ordered water structure at the inner Helmholtz plane. The well-ordered hydrogen-bond network, characterized by shorter hydrogen bonds, enables efficient electron and proton transfer to the surface. As shown in Fig. 3b, the intensity of the Au–H bond exhibits a similar trend to that of the dangling O–H bond, suggesting that hydration water with dangling O–H bonds (unbound hydrogen bonds) serves as a proton donor. According to previously reported electrostatic mechanisms, the intensity of the dangling O–H bond increases with a negative potential shift, which is attributed to the strong dipole of water or solvated cations^8,9,53,54^. However, in Ar-saturated electrolyte, the intensity of the dangling O–H bond (Supplementary Fig. 19b) mirrors the trend of interfacial water ordering, implying that dangling O–H bonds originate from ${{\rm{CO}}}_{3}^{2-}$-induced, ordered hydration water molecules. Simulations (Fig. 2b) further reveal that the much lower Gibbs free energies of proton migration from the ${{\rm{CO}}}_{3}^{2-}$ hydration layer to the electrode, and the solvated K^+^, indirectly promote water ordering via stabilizing hydrated carbonates near the Au surface. Therefore, we propose that at potentials more cathodic than –0.8 V_RHE_, the Volmer step (the rate-limiting step of HER) is facilitated due to the low-barrier delivery of electrons and protons to the Au surface enhanced by the ${{\rm{CO}}}_{3}^{2-}$-induced, ordered interfacial water layer, thus suppressing the CO_2_RR.

We explored the mechanism of water decomposition mediated by hydrated ${{\rm{CO}}}_{3}^{2-}$ and proton delivery to the Au surface by computing the thermodynamics of (bi)carbonate intermediates with two hydration water molecules (*n* = 2 H_2_O; Supplementary Fig. 10) on Au(100). The thermoneutral charge transfer from ${{\rm{CO}}}_{3}^{2-}$ to water, generating ${{\rm{CO}}}_{3}^{\bullet -}$, enhances the decomposition of interfacial water (intermediate 2 in Fig. 3c). Subsequently, the delivered H migrates to the surface via a Grotthuss-like mechanism with the carbonate radical acting as relay (intermediates 3 and 4)^55^. Moreover, the charge density difference plotted in Fig. 3d for ${{\rm{CO}}}_{3}^{2-}$ and ${{\rm{CO}}}_{3}^{\bullet -}$ reveals a higher depletion of electrons (black box) towards H_2_O for the carbonate anion radical, thereby favouring charge transfer to water. We also computed the reaction profiles from hydrated ${{\rm{CO}}}_{3}^{2-}$ (*n* = 2 H_2_O) to H_2_ generation on Au(100), demonstrating a downhill path for the HER at *U* = –0.8 V_RHE_ (Fig. 3e). Plotting these profiles alongside the alternative reduction of ${{\rm{CO}}}_{3}^{2-}$ to HCHO reveals that while at *U* = –0.6 V_RHE_ the *HCO_2_ formation can compete with the Heyrovsky step, at potentials more cathodic than –0.8 V_RHE_, HER will dominate (Supplementary Fig. 20). These potential windows of reactivity align well with reported selectivity potential regimes^56^ and with our DEMS detection of ${{\rm{H}}}_{2}^{+}$ and CHO^+^ (Fig. 1c), providing key mechanistic insights into the competition between CO_2_RR and HER on Au surfaces.

**Fig. 4:**
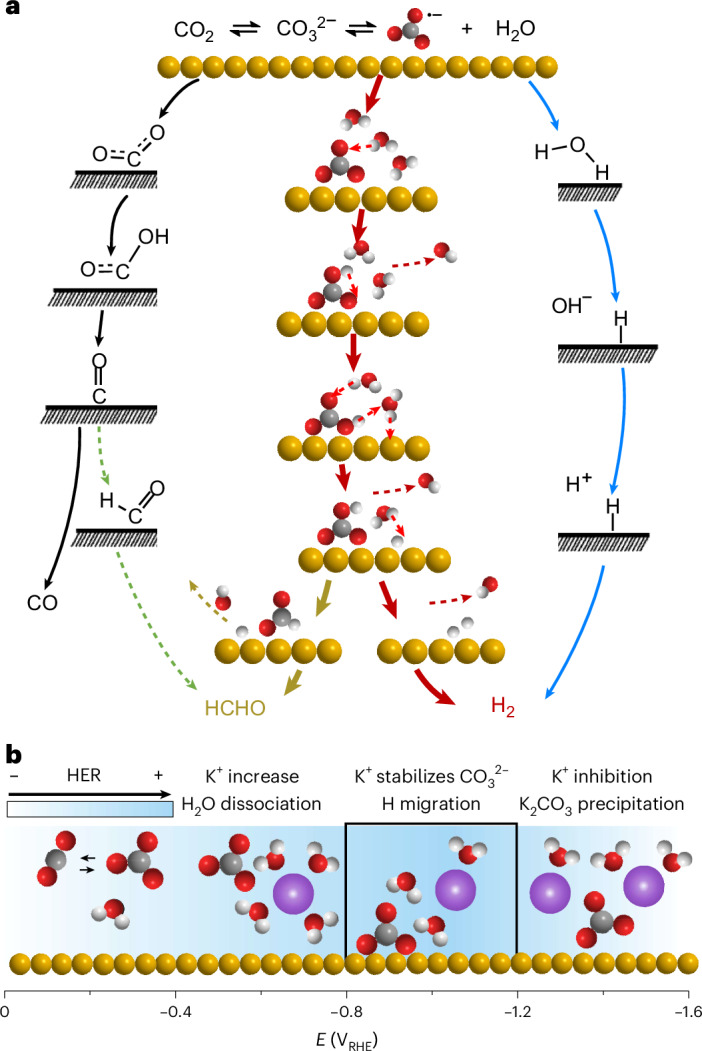
Schematic of catalytic mechanisms on Au surface. **a**, Overview of the reaction pathways for CO_2_ electroreduction to CO (in black), CO_2_ reduction to HCHO (in green), ${{\rm{CO}}}_{3}^{\bullet -}$ reduction to HCHO (in dark yellow), ${{\rm{CO}}}_{3}^{\bullet -}$-facilitated HER (in red) and H_2_O decomposed to H_2_ (in blue). **b**, Illustration of electrolyte effects on HER on the Au surface during CO_2_RR. Au, C, O, H and K^+^ are depicted in yellow, grey, red, white and purple, respectively.

As a summary of the observed reactivity on Au catalysts, the schematic mechanisms illustrating the CO_2_RR, ${{\rm{CO}}}_{3}^{\bullet -}$ reduction and HER are presented in Fig. 4, highlighting the role of the structured hydration shell. In Fig. 4a, the well-established pathway for CO production on Au (highlighted in black) can diverge towards formaldehyde (depicted in green) as the *CO intermediate undergoes further hydrogenation. In turn, water structuring around carbonate enhances a H^+^ delivery mechanism (in red) from ${{\rm{CO}}}_{3}^{\bullet -}$, facilitating the formation of Au–H bonds and thus leading to the dominant HER (in red), and alternatively (in yellow) to ${{\rm{CO}}}_{3}^{\bullet -}$ reduction to HCHO. A comprehensive view of electrolyte effects on the HER during CO_2_RR is illustrated in Fig. 4b. At low overpotential, Au–H bond formation mainly results from H_2_O decomposition, with minimal influence from K^+^ and ${{\rm{CO}}}_{3}^{2-}$ impacts due to their lower concentration near the Au surface. With a negative potential sweep, K^+^ and ${{\rm{CO}}}_{3}^{2-}$ begin to accumulate on the Au surface, promoting the first step of HER by increasing the ordering of interfacial water. During this process, cation effects appear to dominate because of the relatively lower concentration of ${{\rm{CO}}}_{3}^{2-}$. With the specific adsorption of carbonates detected as the potential approaches –0.8 V_RHE_, the ${{\rm{CO}}}_{3}^{2-}$-induced, ordered hydration water layer facilitates proton migration to the Au surface through a well-connected hydrogen-bond network. Solvated K^+^ clusters do not directly facilitate the proton delivery to the Au surface but stabilize hydrated ${{\rm{CO}}}_{3}^{2-}$ near the electrode. At higher potentials (<−1.2 V_RHE_), the loss of ${{\rm{CO}}}_{3}^{2-}$ adsorption and K^+^ precipitation lead to low HER activity, due to hindered proton migration to the Au surface.

## Discussion

This in situ study revealed the intricate interplay between carbon and proton sources within the microenvironment of bicarbonate electrolytes and highlighted the inherent impact of the local interfacial water structure on the competing mechanisms of CO_2_RR and HER on gold electrocatalysts. Using vibrational spectroscopy (ATR-SEIRAS) and electrochemical spectrometry (DEMS) alongside DFT, we found that carbonate molecules adsorbed on the Au surface contribute to an enhancement of the structural order of the surrounding water hydration layers. Carbonate anion radicals $\left({{\rm{CO}}}_{3}^{\bullet -}\right)$, formed from hydrated carbonate, can act as a carbon source and be reduced into either HCHO or CO at potentials near –0.6 V_RHE_. At higher cathodic potentials, CO_2_RR is suppressed by HER, facilitated by the rapid delivery of protons from ordered interfacial hydration networks mediated by the ${{\rm{CO}}}_{3}^{\bullet -}$ relay. Molecular-level evidence supports water as the primary proton donor for the Volmer step.

Our findings show that carbonate molecules modify the local structure of EDLs, and along with the identification of carbonate radicals as proton relays, they highlight the importance of explicitly considering interfacial solution structures and related specific ion effects in current models of electrochemical interfaces. The detection of aldehydes prompts re-evaluating established reaction mechanisms on gold electrocatalysts, underscoring that reactants can alternatively originate from in situ transient electrolyte species. Incorporating these effects into theoretical frameworks will enhance their predictive accuracy and validity. The molecular-level insights provided are broadly relevant to all electrochemical systems in aqueous electrolytes where carbonates are commonly present. They will aid in rationally improving the selectivity and activity of electrocatalytic reactions and controlling the properties of electrochemical interfaces by actively steering interfacial solution structures, with various applications, including electrochemical technologies, photochemistry and energy conversion.

## Methods

### Online DEMS measurements

A home-built probe-type DEMS system, using a commercial mass spectrometer (Hiden-40PR), was employed to monitor both the volatile products and the consumed CO_2_ during CO_2_RR. The response time of online DEMS is below 1 s. The details of the DEMS set-up were reported previously^57^. A probe, covered by a porous membrane, was positioned at a distance of 10 µm from the working electrode, regulated by a micrometre screw gauge and measured by a digital microscope. During DEMS measurements, linear sweep voltammetry scans were conducted from 0.3 V_RHE_ to –1.0 V_RHE_ at a scan rate of 2 mV s^−1^, with an electrolyte flow rate of 1.25 ml min^−1^, while ionic currents were recorded by the mass spectrometer. DEMS experiments with isotopic labelling were carried out under identical conditions, except for the substitution of D_2_O (100 at.% D; Carl Roth) in place of H_2_O for preparing the KHCO_3_ electrolyte. For quantitative analysis, the calibration constant K* was defined as K* = *n*MS/*I*_F_, with *n* denoting the number of electrons, MS denoting the mass spectrometric signal intensity and *I*_F_ denoting the faradaic current. Here, a commercial polycrystalline gold disc of 3 mm diameter served as the working electrode. Prior to conducting the electrochemical measurements, the Au electrode underwent successive polishing steps using alumina particles of varying particle sizes, ranging from 1.0 µm to 0.3 µm to 0.05 µm, followed by rinsing with deionized water. Subsequently, the polished Au electrode was activated in 0.5 M H_2_SO_4_ through electrochemical cycling between 0.05 and 1.65 V_RHE_ at a scan rate of 50 mV s^−1^ until a stable cyclic voltammogram was obtained. A reference hydrogen electrode and a high-purity carbon rod as counter electrode were employed for electrochemical tests.

### In situ ATR-SEIRAS measurements

In situ ATR IR measurements were carried out using a Bruker VERTEX 80v Fourier transform IR spectrometer, equipped with a mercury cadmium telluride (MCT) detector cooled by liquid nitrogen, operating at a spectral resolution of 4 cm^−1^ and with both s-polarized and p-polarized IR radiation. The acquisition time for each spectrum was about 8 s with 16 scans (~0.50 s per scan). The working electrode was a Au-film-coated Si wafer, serving as the optical window within a self-designed spectro-electrochemical cell, with a carbon rod as the counter electrode and a RHE (Mini-HydroFlex, Gaskatel) as the reference electrode. The preparation of the Au-coated Si wafer followed a chemical method outlined in previous work^57,58^. Before depositing Au, the reflection surface of the Si wafer was polished with chemical grade alumina powder (1.0, 0.3 and 0.05 μm) until totally hydrophobic, and then cleaned several times in sonication with acetone (≥99.5%, American Chemical Society (ACS) reagent) and deionized water. After that, the Si wafer was dried by argon flow and immersed in common piranha solution (3:1 in volume, 95–98% H_2_SO_4_ and 35% H_2_O_2_; Carl Roth) for 30 min to remove the organic residues, cleaned in deionized water and dried by argon flow. Subsequently, the reflection plane of the Si wafer was contacted with 40% NH_4_F (≥98%, ACS reagent; Carl Roth) solution for 90 s to terminate it with hydrogen. The hydrogen-terminated surface was dried by argon flow and immediately immersed into a mixture of 2 ml Au plating solution^59^ and 30 µl 40% HF solution (Merck Group) at 55–60 °C for 5 min. The good conductivity of the Au film featured an ohmic resistance below 3 Ω. The Si wafer (<111>; Siegert Wafer) featured a thickness of 0.5 mm to extend the detectable wave number to below 1,000 cm^−1^ by minimizing the interfering Si prism absorption signal. Electrochemical measurements were performed using a commercial potentiostat (Autolab, model PGSTAT302N) connected to the Fourier transform IR set-up, facilitating automated triggering of spectroscopic measurements. In both Ar-saturated and CO_2_-saturated KHCO_3_ electrolyte, the reference spectrum was recorded at 0.3 V_RHE_, a potential within the charging/discharging region devoid of faradaic reactions. The isotopic labelling ATR IR measurements were carried out in Ar-saturated electrolyte by replacing KHCO_3_ by KH^13^CO_3_ (98 at.% ^13^C; Sigma-Aldrich), and replacing H_2_O with D_2_O (100 at.% D; Carl Roth). Inherently, the in situ spectra accurately depict potential-dependent intermediates during reactions, minimizing deduction from preselected reference spectra. The IR signal intensity varies between samples due to the configuration of Au islands on the Au film (that is, the distinct local roughness), where factors such as the island dimensions and aspect ratios as well as the relative distances between islands influence the electromagnetic enhancement effect. Thus, for the analysis of potential-dependent IR spectra, individual datasets from a series of repeated measurements are presented rather than averaged data. Each ATR-SEIRAS dataset was measured independently three times, showing a consistent potential-dependent trend in the relative intensities of the observed fingerprints.

### Computational details

All models were obtained by DFT simulations using Vienna Ab initio Simulation Package (VASP)^60^. The exchange functional used was the Perdew–Burke–Ernzerhof^61^. Dispersion was included through the DFT-D2 method^60,62^, with our reparametrized C_6_ coefficients for Au atoms^63^. Inner electrons were represented through projector augmented-wave pseudopotentials^64,65^, and the monoelectronic states for the valence electrons were expanded as plane waves with a kinetic energy cut-off of 450 eV (ref. ^60^). Mechanistic studies were performed on Au(100) and Au(111) asymmetric surface models, and thus the dipole correction was applied^66^. The Brillouin zone was sampled by a Γ-centred k-point mesh from the Monkhorst–Pack method^67^ with a reciprocal grid size smaller than 0.03 × 2π Å^−1^ and a three-dimensional periodic cubic box. For all the investigated systems, structures were relaxed using convergence criteria of 0.03 eV Å^−1^ and 10^−5^ eV for the ionic and electronic steps, respectively. The VASPsol++ framework^68^ was used in the grand canonical DFT simulations to account for the potential and the electrolyte effects. For these specific cases, the vacuum along the *z* direction was increased up to 26 Å. The ion radius of the implicit K^+^ hydration shell was set to 3.30 Å (refs. ^69,70^), and the concentration of cations was assumed to be 1 M. Implicit potentials of *U* = 0, –0.4, –0.6, –0.8 and –1.0 V_RHE_ were applied by updating the number of electrons in each geometric optimization step, considering that the Fermi level of the surface is referenced to the bulk electrolyte^71^. Gibbs free energies of reaction intermediates were obtained using the gas-phase energies of CO_2_, H_2_O and H_2_/H^+^ as thermodynamic sinks (equations (3)–(6)). The CHE was used to obtain the relative energy between H^+^ and H_2_ at *U* = 0 V_RHE_ (equation (7))^72,73^.

### DFT structural models

Mechanistic studies were performed on Au(100) and Au(111) surface models on (3 × 3) and (4 × 4) supercells, respectively, with five layers, with the three lowermost layers fixed at bulk positions and the rest fully relaxed. The vacuum thickness between periodic repetitions in the *z* direction accounted for at least 10 Å. Simulations including cations in their explicit hydration layer were modelled as K^+^(H_2_O)_4_, using the structures previously computed by our group^18^. The carbonate anion radical bidentate adsorption (*η*_O,O_) was simulated on both surfaces starting from the most stable ${{\rm{CO}}}_{3}^{\bullet -}$ reported structure^52^ and then fully relaxing it, computing it with fixed C–O^•^ bond distance (*d*_C–O•_ = 1.37 Å) and computing it with all fixed distances. Then all three cases were benchmarked using the magnetization values as an indicator of proper electronic convergence as a radical. Numerical frequencies were computed by finite displacements with a step size of ±0.015 Å. By fixing at least the C–O^•^ bond distance, the magnetization values showed the electronic structure of a radical (Supplementary Table 2), and when the whole carbonate radical anion geometry was fixed, frequencies shifted closer to the 1,100 cm^−1^ IR feature assigned to the C–O symmetric stretching band (*ν*_1_; Supplementary Fig. 9). Carbonates are known to form stable clusters with one to six H_2_O molecules, which breaks the anion *D*_3*h*_ symmetry and blueshifts the frequencies^52,74,75^. Therefore, ${{\rm{CO}}}_{3}^{\bullet -}$ structures featuring a hydration of *n*H_2_O molecules (*n* = 0, 1, 2 and 3) were also computed to represent the interfacial water hydration, as it has been shown that at least two water molecules are required to properly obtain their solvated water HOMO energy levels^44^.

### Energies of intermediates

To obtain standard Gibbs free energies following the CHE approach^72^, zero-point energy (*E*_ZPE_) and entropy (*S*) contributions were added to VASP-derived potential energies (*E*; equation (1)). The zero-point energy contribution was obtained from the vibrational contribution to the internal thermal energy (equation (2)). Vibrational frequencies (*ν*_*K*_, where *K* refers to a variable from the summatory, which accounts for all the computed frequencies) were taken from VASP. Frequencies below 24.80 meV (200 cm^−1^) were made equal to this number to avoid unphysical entropic contributions. The entropy contribution was computed as the vibrational entropy (*S*_*ν*_; equation (3)), for which the standard entropy value was taken from the literature as the experimental entropy in the gas phase at standard conditions^76^.

1 $${G}^{0}={E}_{\mathrm{VASP}}+{E}_{\mathrm{ZPE}}-{TS}$$

2 $${E}_{\mathrm{ZPE}}=\frac{1}{2}{k}_{{\rm{B}}}{\sum }_{K}{\theta }_{\nu ,K}$$

3 $$S={S}_{\nu }={k}_{{\rm{B}}}\mathop{\sum }\limits_{K}\left(\frac{{\theta }_{\nu ,K}/T}{{{\rm{e}}}^{{\theta }_{\nu ,K}/T}-1}-\mathrm{ln}\left(1-{{\rm{e}}}^{-{\theta }_{\nu ,K}/T}\right)\right)$$

where *G*^0^ is the Gibbs free energy at normal conditions, *E*_VASP_ is the potential energy computed by DFT in the VASP software, *T* is temperature, *k*_B_ is the Boltzmann constant and *θ*_*v*,*K*_ refers to each of the vibrational frequencies after uploading the values for those below 24.8 mV. The CHE was used to obtain the relative energy of H^+^ from H_2_ at *U* = 0 V_RHE_ (equation (4))^72^.

4 $${E}_{{{\rm{H}}}^{+}}=\frac{1}{2}{E}_{{{\rm{H}}}_{2}}$$

Potential energies of reaction intermediates were obtained using CO_2_, H_2_O and H_2_/H^+^ as thermodynamic references (equations (5)–(7)). The factor Δe^*−*^, an increment of electron accounting in the use of CHE due to the charging of a species, was applied to intermediates with an electron-enhanced adsorption, for example, ${\ast\atop}{\rm{C}}{{\rm{O}}}_{2}^{-}$ (equation (8)). The factor ∆*Q*_B_, representing the difference in Bader charges, uses Bader charges of intermediates to add a polarization term as a correction to the DFT energy (*E*_DFT_)^77^.

5 $$\begin{array}{c}{\rm{x}}{\mathrm{CO}}_{2}+* +({\rm{z}}-2{\rm{x}}){{\rm{H}}}_{2}{\rm{O}}+(4{\rm{x}}+{\rm{y}}-2{\rm{z}}){{\rm{H}}}^{+}+(4{\rm{x}}+{\rm{y}}-2{\rm{z}}){{\rm{e}}}^{-}\\ +{\mathrm{\varDelta e}}^{-}\to * {\mathrm{C}}_{{\rm{x}}}{{\rm{H}}}_{{\rm{y}}}{{\rm{O}}}_{{\rm{z}}}\end{array}\,$$

6 $$\begin{array}{c}{E}_{* {\mathrm{C}}_{x}{{\rm{H}}}_{y}{{\rm{O}}}_{z}}={E}_{\mathrm{DFT},* {\mathrm{C}}_{x}{{\rm{H}}}_{y}{{\rm{O}}}_{z}}-{E}_{* }-(x){E}_{{\mathrm{CO}}_{2}}-(z-2x){E}_{{{\rm{H}}}_{2}{\rm{O}}}\\ -(4x+y-2z){E}_{{{\rm{H}}}^{+}}+{n}_{{{\rm{e}}}^{-}}U\end{array}\,$$

7 $${n}_{{{\rm{e}}}^{-}}=\left(4x+y-2z\right)+\Delta {{\rm{e}}}^{-}+\Delta {Q}_{{\rm{B}}}$$

8 $$* +{{\rm{C}}\mathrm{O}}_{2}+{{\rm{e}}}^{-}\to * \mathrm{C}{\mathrm{O}}_{2}^{-}$$

Asterisks refers to the potential energy of the clean surface without adsorbates. The energies of Au–H formation by the carbonate-enhanced H^+^ delivery mechanism (Fig. 2b) are obtained considering the decomposition of hydrated ${{\rm{CO}}}_{3}^{\bullet -}$ (*n* = 1, 2 and 3) interfacial water. For this, equation (11) was used, which combines equations (9) and (10) to include the contribution of the water equilibrium constant *K*_w_ (equation (12)).

9 $$* +{\mathrm{CO}}_{3}^{{\rm{\bullet }}-}{\mathrm{n}}{{\rm{H}}}_{2}{\rm{O}}+{{\rm{e}}}^{-}\leftrightarrow * {\rm{H}}+{\mathrm{CO}}_{3}^{{\rm{\bullet }}-}\left(n-1\right){{\rm{H}}}_{2}{\rm{O}}+{\mathrm{OH}}^{-}$$

10 $${{\rm{H}}}^{+}+{{\rm{OH}}}^{-}\leftrightarrow {{\rm{H}}}_{2}{\rm{O}}$$

11 $$\Delta G={G}_{\mathrm{DFT},* \mathrm{H}+{\mathrm{CO}}_{3}^{{\rm{\bullet }}-}\left(n-1\right){{\rm{H}}}_{2}{\rm{O}}}^{0}+{G}_{\mathrm{C}{\mathrm{O}}_{2}}^{2}-{G}_{\mathrm{DFT},* +{\mathrm{CO}}_{3}^{{\rm{\bullet }}-}{{\mathrm{n}}{\rm{H}}}_{2}{\rm{O}}}^{0}-{G}_{{{\rm{H}}}^{+}}^{0}-{\Delta G}_{{\rm{w}}}^{0}$$

12 $${\Delta G}_{{\rm{w}}}^{0}=-{k}_{{\rm{B}}}T\mathrm{ln}{K}_{{\rm{w}}}$$

The Gibbs free energy of charge transfer from carbonate to water, generating a carbonate ion radical (equation (13)), was computed using Gaussian v.09 (ref. ^78^) at the M06-2X level^79^ and with the AUG-cc-pVTZ basis sets for all atoms. To represent aqueous media, water was used as the implicit solvent with the solvent model based on density (SMD) approach^80^. The optimized structures were characterized as minima of the corresponding potential energy surfaces by means of the diagonalized Hessian matrices’ eigenvalues.

13 $${{\rm{CO}}}_{3}^{2-}+{{\rm{OH}}}^{{\rm{\bullet }}}\leftrightarrow {{\rm{CO}}}_{3}^{{\rm{\bullet }}-}+{{\rm{OH}}}^{-}$$

## Online content

Any methods, additional references, Nature Portfolio reporting summaries, source data, extended data, supplementary information, acknowledgements, peer review information; details of author contributions and competing interests; and statements of data and code availability are available at 10.1038/s41557-025-01977-8.

## Supplementary information

Supplementary Information Supplementary Figs. 1–20, Tables 1–3, Notes 1–5 and Discussion.

Supplementary Data 1 Data shown in Supplementary Figs. 4–20.

## Source data

Source Data Fig. 1 Source data.

Source Data Fig. 2 Source data.

Source Data Fig. 3 Source data.

## Data Availability

Supporting DFT datasets are available in ioChem-BD^81^ at 10.19061/iochem-bd-1-305. All other raw data are available from the corresponding author upon reasonable request. Source data are provided with this paper.

## References

[1] Birdja, Y. Y. et al. Advances and challenges in understanding the electrocatalytic conversion of carbon dioxide to fuels. *Nat. Energy* **4**, 732–745 (2019).

[2] Nitopi, S. et al. Progress and perspectives of electrochemical CO_2_ reduction on copper in aqueous electrolyte. *Chem. Rev.* **119**, 7610–7672 (2019). 31117420 10.1021/acs.chemrev.8b00705

[3] Martín, A. J., Larrazábal, G. O. & Pérez-Ramírez, J. Towards sustainable fuels and chemicals through the electrochemical reduction of CO_2_: lessons from water electrolysis. *Green Chem.* **17**, 5114–5130 (2015).

[4] Monteiro, M. C., Philips, M. F., Schouten, K. J. P. & Koper, M. T. Efficiency and selectivity of CO_2_ reduction to CO on gold gas diffusion electrodes in acidic media. *Nat. Commun.* **12**, 4943 (2021). 34400626 10.1038/s41467-021-24936-6 PMC8368099

[5] Ma, H. et al. Direct electroreduction of carbonate to formate. *J. Am. Chem. Soc.* **145**, 24707–24716 (2023). 37924283 10.1021/jacs.3c08079 PMC10655187

[6] Vass, Á, Kormányos, A., Kószó, Z., Endrodi, B. & Janáky, C. Anode catalysts in CO_2_ electrolysis: challenges and untapped opportunities. *ACS Catal.* **12**, 1037–1051 (2022). 35096466 10.1021/acscatal.1c04978 PMC8787754

[7] Xu, Y., Ma, Y.-B., Gu, F., Yang, S.-S. & Tian, C.-S. Structure evolution at the gate-tunable suspended graphene–water interface. *Nature* **621**, 506–510 (2023). 37648858 10.1038/s41586-023-06374-0

[8] Li, C.-Y. et al. In situ probing electrified interfacial water structures at atomically flat surfaces. *Nat. Mater.* **18**, 697–701 (2019). 31036960 10.1038/s41563-019-0356-x

[9] Wang, Y.-H. et al. In situ Raman spectroscopy reveals the structure and dissociation of interfacial water. *Nature* **600**, 81–85 (2021). 34853456 10.1038/s41586-021-04068-z

[10] Li, P. et al. Hydrogen bond network connectivity in the electric double layer dominates the kinetic pH effect in hydrogen electrocatalysis on Pt. *Nat. Catal.* **5**, 900–911 (2022).

[11] Strmcnik, D. et al. Improving the hydrogen oxidation reaction rate by promotion of hydroxyl adsorption. *Nat. Chem.* **5**, 300–306 (2013). 23511418 10.1038/nchem.1574

[12] Wang, T. et al. Enhancing oxygen reduction electrocatalysis by tuning interfacial hydrogen bonds. *Nat. Catal.* **4**, 753–762 (2021).

[13] Goyal, A. & Koper, M. T. The interrelated effect of cations and electrolyte pH on the hydrogen evolution reaction on gold electrodes in alkaline media. *Angew. Chem. Int. Ed.* **60**, 13452–13462 (2021). 10.1002/anie.202102803 PMC8252582 33769646

[14] Goyal, A. & Koper, M. Understanding the role of mass transport in tuning the hydrogen evolution kinetics on gold in alkaline media. *J. Chem. Phys.* **155**, 134705 (2021). 10.1063/5.0064330 34624997

[15] Monteiro, M. C., Goyal, A., Moerland, P. & Koper, M. T. Understanding cation trends for hydrogen evolution on platinum and gold electrodes in alkaline media. *ACS Catal.* **11**, 14328–14335 (2021). 34888121 10.1021/acscatal.1c04268 PMC8650008

[16] Zhang, Z.-M. et al. Probing electrolyte effects on cation-enhanced CO_2_ reduction on copper in acidic media. *Nat. Catal.* **7**, 807–817 (2024).

[17] Liu, X. & Koper, M. T. Tuning the interfacial reaction environment for CO_2_ electroreduction to CO in mildly acidic media. *J. Am. Chem. Soc.* **146**, 5242–5251 (2024). 38350099 10.1021/jacs.3c11706 PMC10910518

[18] Monteiro, M. C. O. et al. Absence of CO_2_ electroreduction on copper, gold and silver electrodes without metal cations in solution. *Nat. Catal.* **4**, 654–662 (2021).

[19] Goyal, A., Marcandalli, G., Mints, V. A. & Koper, M. T. Competition between CO_2_ reduction and hydrogen evolution on a gold electrode under well-defined mass transport conditions. *J. Am. Chem. Soc.* **142**, 4154–4161 (2020). 32041410 10.1021/jacs.9b10061 PMC7059182

[20] Zhang, B. A., Ozel, T., Elias, J. S., Costentin, C. & Nocera, D. G. Interplay of homogeneous reactions, mass transport, and kinetics in determining selectivity of the reduction of CO_2_ on gold electrodes. *ACS Central Sci.* **5**, 1097–1105 (2019). 10.1021/acscentsci.9b00302 PMC6598161 31263769

[21] Wuttig, A., Yoon, Y., Ryu, J. & Surendranath, Y. Bicarbonate is not a general acid in Au-catalyzed CO_2_ electroreduction. *J. Am. Chem. Soc.* **139**, 17109–17113 (2017). 28978199 10.1021/jacs.7b08345

[22] Cave, E. R. et al. Electrochemical CO_2_ reduction on Au surfaces: mechanistic aspects regarding the formation of major and minor products. *Phys. Chem. Chem. Phys.* **19**, 15856–15863 (2017). 28585950 10.1039/c7cp02855e

[23] Zhu, W. et al. Monodisperse Au nanoparticles for selective electrocatalytic reduction of CO_2_ to CO. *J. Am. Chem. Soc.* **135**, 16833–16836 (2013). 24156631 10.1021/ja409445p

[24] Chen, Y., Li, C. W. & Kanan, M. W. Aqueous CO_2_ reduction at very low overpotential on oxide-derived Au nanoparticles. *J. Am. Chem. Soc.* **134**, 19969–19972 (2012). 23171134 10.1021/ja309317u

[25] Goodpaster, J. D., Bell, A. T. & Head-Gordon, M. Identification of possible pathways for C–C bond formation during electrochemical reduction of CO_2_: new theoretical insights from an improved electrochemical model. *J. Phys. Chem. Lett.* **7**, 1471–1477 (2016). 27045040 10.1021/acs.jpclett.6b00358

[26] Montoya, J. H., Shi, C., Chan, K. & Nørskov, J. K. Theoretical insights into a CO dimerization mechanism in CO_2_ electroreduction. *J. Phys. Chem. Lett.* **6**, 2032–2037 (2015). 26266498 10.1021/acs.jpclett.5b00722

[27] Wang, X. L. et al. Mechanistic reaction pathways of enhanced ethylene yields during electroreduction of CO_2_–CO co-feeds on Cu and Cu-tandem electrocatalysts. *Nat. Nanotechnol.* **14**, 1063–1070 (2019). 31591526 10.1038/s41565-019-0551-6

[28] Zhu, S., Jiang, B., Cai, W.-B. & Shao, M. Direct observation on reaction intermediates and the role of bicarbonate anions in CO_2_ electrochemical reduction reaction on Cu surfaces. *J. Am. Chem. Soc.* **139**, 15664–15667 (2017). 29058890 10.1021/jacs.7b10462

[29] Dunwell, M. et al. The central role of bicarbonate in the electrochemical reduction of carbon dioxide on gold. *J. Am. Chem. Soc.* **139**, 3774–3783 (2017). 28211683 10.1021/jacs.6b13287

[30] Marcandalli, G., Villalba, M. & Koper, M. T. M. The importance of acid–base equilibria in bicarbonate electrolytes for CO_2_ electrochemical reduction and CO reoxidation studied on Au(*hkl*) electrodes. *Langmuir* **37**, 5707–5716 (2021). 33913319 10.1021/acs.langmuir.1c00703 PMC8154874

[31] Wuttig, A., Yaguchi, M., Motobayashi, K., Osawa, M. & Surendranath, Y. Inhibited proton transfer enhances Au-catalyzed CO_2_-to-fuels selectivity. *Proc. Natl Acad. Sci. USA* **113**, E4585–E4593 (2016). 27450088 10.1073/pnas.1602984113 PMC4987813

[32] Calle-Vallejo, F., Loffreda, D., Koper, M. T. M. & Sautet, P. Introducing structural sensitivity into adsorption–energy scaling relations by means of coordination numbers. *Nat. Chem.* **7**, 403–410 (2015). 25901818 10.1038/nchem.2226

[33] Mezzavilla, S., Horch, S., Stephens, I. E., Seger, B. & Chorkendorff, I. Structure sensitivity in the electrocatalytic reduction of CO_2_ with gold catalysts. *Angew. Chem. Int. Ed.* **58**, 3774–3778 (2019). 10.1002/anie.201811422 30673156

[34] Monteiro, M. C. et al. Time-resolved local pH measurements during CO_2_ reduction using scanning electrochemical microscopy: buffering and tip effects. *JACS Au* **1**, 1915–1924 (2021). 34849509 10.1021/jacsau.1c00289 PMC8611793

[35] Seong, H. et al. Atomically precise gold nanoclusters as model catalysts for identifying active sites for electroreduction of CO_2_. *Angew. Chem. Int. Ed.* **60**, 14563–14570 (2021). 10.1002/anie.202102887 33877721

[36] Ringe, S. et al. Understanding cation effects in electrochemical CO_2_ reduction. *Energy Environ. Sci.* **12**, 3001–3014 (2019).

[37] Malkani, A. S., Anibal, J. & Xu, B. Cation effect on interfacial CO_2_ concentration in the electrochemical CO_2_ reduction reaction. *ACS Catal.* **10**, 14871–14876 (2020).

[38] Monteiro, M. C. O., Dattila, F., López, N. & Koper, M. T. M. The role of cation acidity on the competition between hydrogen evolution and CO_2_ reduction on gold electrodes. *J. Am. Chem. Soc.* **144**, 1589–1602 (2022). 34962791 10.1021/jacs.1c10171 PMC8815072

[39] Chen, C., Zhang, B., Zhong, J. & Cheng, Z. Selective electrochemical CO_2_ reduction over highly porous gold films. *J. Mater. Chem. A* **5**, 21955–21964 (2017).

[40] Yoon, Y., Hall, A. S. & Surendranath, Y. Tuning of silver catalyst mesostructure promotes selective carbon dioxide conversion into fuels. *Angew. Chem. Int. Ed.* **55**, 15282–15286 (2016). 10.1002/anie.201607942 27862743

[41] Clark, E. L. et al. Standards and protocols for data acquisition and reporting for studies of the electrochemical reduction of carbon dioxide. *ACS Catal.* **8**, 6560–6570 (2018).

[42] Clark, E. L. & Bell, A. T. Direct observation of the local reaction environment during the electrochemical reduction of CO_2_. *J. Am. Chem. Soc.* **140**, 7012–7020 (2018). 29756446 10.1021/jacs.8b04058

[43] Arihara, K., Kitamura, F., Ohsaka, T. & Tokuda, K. Characterization of the adsorption state of carbonate ions at the Au(111) electrode surface using in situ IRAS. *J. Electroanal. Chem.* **510**, 128–135 (2001).

[44] Bellarosa, L., Castillo, J. M., Vlugt, T., Calero, S. & López, N. On the mechanism behind the instability of isoreticular metal–organic frameworks (irmofs) in humid environments. *Chem. Eur. J.* **18**, 12260–12266 (2012). 22907782 10.1002/chem.201201212

[45] Wuttig, A. et al. Tracking a common surface-bound intermediate during CO_2_-to-fuels catalysis. *ACS Central Sci.* **2**, 522–528 (2016). 10.1021/acscentsci.6b00155 PMC4999975 27610413

[46] Nørskov, J. K. et al. Trends in the exchange current for hydrogen evolution. *J. Electrochem. Soc.* **152**, J23–J26 (2005).

[47] Zhao, S., Jin, R. & Jin, R. Opportunities and challenges in CO_2_ reduction by gold- and silver-based electrocatalysts: from bulk metals to nanoparticles and atomically precise nanoclusters. *ACS Energy Lett.* **3**, 452–462 (2018).

[48] Nichols, R. J. & Bewick, A. Spectroscopic identification of the adsorbed intermediate in hydrogen evolution on platinum. *J. Electroanal. Chem. Interfacial Electrochem.* **243**, 445–453 (1988).

[49] Tian, Z.-Q., Ren, B., Chen, Y.-X., Zou, S.-Z. & Mao, B.-W. Probing electrode/electrolyte interfacial structure in the potential region of hydrogen evolution by Raman spectroscopy. *J. Chem. Soc. Faraday Trans.* **92**, 3829–3838 (1996).

[50] Peremans, A. & Tadjeddine, A. Vibrational spectroscopy of electrochemically deposited hydrogen on platinum. *Phys. Rev. Lett.* **73**, 3010–3013 (1994). 10057259 10.1103/PhysRevLett.73.3010

[51] Pan, M., Pozun, Z. D., Yu, W.-Y., Henkelman, G. & Mullins, C. B. Structure revealing H/D exchange with co-adsorbed hydrogen and water on gold. *J. Phys. Chem. Lett.* **3**, 1894–1899 (2012). 26292010 10.1021/jz3007707

[52] Zilberg, S., Mizrahi, A., Meyerstein, D. & Kornweitz, H. Carbonate and carbonate anion radicals in aqueous solutions exist as and respectively: the crucial role of the inner hydration sphere of anions in explaining their properties. *Phys. Chem. Chem. Phys.* **20**, 9429–9435 (2018). 29565065 10.1039/C7CP08240A

[53] Tong, Y., Lapointe, F., Thämer, M., Wolf, M. & Campen, R. K. Hydrophobic water probed experimentally at the gold electrode/aqueous interface. *Angew. Chem. Int. Ed.* **56**, 4211–4214 (2017). 10.1002/anie.201612183 28300334

[54] Montenegro, A. et al. Asymmetric response of interfacial water to applied electric fields. *Nature* **594**, 62–65 (2021). 34079138 10.1038/s41586-021-03504-4

[55] Fischer, S. A. & Gunlycke, D. Analysis of correlated dynamics in the Grotthuss mechanism of proton diffusion. *J. Phys. Chem. B* **123**, 5536–5544 (2019). 31180658 10.1021/acs.jpcb.9b02610

[56] Marcandalli, G., Goyal, A. & Koper, M. T. M. Electrolyte effects on the faradaic efficiency of CO_2_ reduction to CO on a gold electrode. *ACS Catal.* **11**, 4936–4945 (2021). 34055454 10.1021/acscatal.1c00272 PMC8154322

[57] Zhou, Y.-W. et al. Probing the enhanced methanol electrooxidation mechanism on platinum-metal oxide catalyst. *Appl. Catal. B* **280**, 119393 (2021).

[58] Jiang, T.-W. et al. Spectrometric study of electrochemical CO_2_ reduction on Pd and Pd-B electrodes. *ACS Catal.* **11**, 840–848 (2021).

[59] Miyake, H., Ye, S. & Osawa, M. Electroless deposition of gold thin films on silicon for surface-enhanced infrared spectroelectrochemistry. *Electrochem. Commun.* **4**, 973–977 (2002).

[60] Kresse, G. & Furthmüller, J. Efficient iterative schemes for *ab initio* total-energy calculations using a plane-wave basis set. *Phys. Rev. B* **54**, 11169–11186 (1996). 10.1103/physrevb.54.11169 9984901

[61] Perdew, J. P., Burke, K. & Ernzerhof, M. Generalized gradient approximation made simple. *Phys. Rev. Lett.* **77**, 3865–3868 (1996). 10062328 10.1103/PhysRevLett.77.3865

[62] Grimme, S. Semiempirical GGA-type density functional constructed with a long-range dispersion correction. *J. Comput. Chem.* **27**, 1787–1799 (2006). 16955487 10.1002/jcc.20495

[63] Almora-Barrios, N., Carchini, G., Błoński, P. & Lopez, N. Costless derivation of dispersion coefficients for metal surfaces. *J. Chem. Theory Comput.* **10**, 5002–5009 (2014). 26584383 10.1021/ct5006467

[64] Blöchl, P. E. Projector augmented-wave method. *Phys. Rev. B* **50**, 17953–17979 (1994). 10.1103/physrevb.50.17953 9976227

[65] Kresse, G. & Joubert, D. From ultrasoft pseudopotentials to the projector augmented-wave method. *Phys. Rev. B* **59**, 1758–1775 (1999).

[66] Makov, G. & Payne, M. C. Periodic boundary conditions in *ab initio* calculations. *Phys. Rev. B* **51**, 4014–4022 (1995). 10.1103/physrevb.51.4014 9979237

[67] Monkhorst, H. J. & Pack, J. D. Special points for Brillouin-zone integrations. *Phys. Rev. B* **13**, 5188–5192 (1976).

[68] Islam, S., Khezeli, F., Ringe, S. & Plaisance, C. An implicit electrolyte model for plane wave density functional theory exhibiting nonlinear response and a nonlocal cavity definition. *J. Chem. Phys.* **159**, 234117 (2023). 38112507 10.1063/5.0176308

[69] Mancinelli, R., Botti, A., Bruni, F., Ricci, M. & Soper, A. Hydration of sodium, potassium, and chloride ions in solution and the concept of structure maker/breaker. *J. Phys. Chem. B* **111**, 13570–13577 (2007). 17988114 10.1021/jp075913v

[70] Zhuang, D., Riera, M., Zhou, R., Deary, A. & Paesani, F. Hydration structure of Na^+^ and K^+^ ions in solution predicted by data-driven many-body potentials. *J. Phys. Chem. B* **126**, 9349–9360 (2022). 36326071 10.1021/acs.jpcb.2c05674

[71] Lindgren, P., Kastlunger, G. & Peterson, A. A. A challenge to the *G* ∼ 0 interpretation of hydrogen evolution. *ACS Catal.* **10**, 121–128 (2019).

[72] Nørskov, J. K. et al. Origin of the overpotential for oxygen reduction at a fuel-cell cathode. *J. Phys. Chem. B* **108**, 17886–17892 (2004). 39682080 10.1021/jp047349j

[73] Peterson, A. A., Abild-Pedersen, F., Studt, F., Rossmeisl, J. & Nørskov, J. K. How copper catalyzes the electroreduction of carbon dioxide into hydrocarbon fuels. *Energy Environ. Sci.* **3**, 1311–1315 (2010).

[74] Pathak, A. K. & Maity, D. K. Distinctive IR signature of and hydrated clusters: a theoretical study. *J. Phys. Chem. A* **113**, 13443–13447 (2009). 19886648 10.1021/jp907577j

[75] Armstrong, D., Waltz, W. & Rauk, A. Carbonate radical anion—thermochemistry. *Can. J. Chem.* **84**, 1614–1619 (2006).

[76] Lide, D. R. (ed.) *CRC Handbook of Chemistry and Physics* Vol. 85 (CRC Press, 2004).

[77] Pablo-García, S. et al. Mechanistic routes toward C_3_ products in copper-catalysed CO_2_ electroreduction. *Catal. Sci. Technol.* **12**, 409–417 (2022).

[78] Frisch, M. et al. Gaussian v.16 revision A.03 (Gaussian, 2016).

[79] Zhao, Y. & Truhlar, D. G. The M06 suite of density functionals for main group thermochemistry, thermochemical kinetics, noncovalent interactions, excited states, and transition elements: two new functionals and systematic testing of four M06-class functionals and 12 other functionals. *Theor. Chem. Acc.* **120**, 215–241 (2008).

[80] Marenich, A. V., Cramer, C. J. & Truhlar, D. G. Universal solvation model based on solute electron density and on a continuum model of the solvent defined by the bulk dielectric constant and atomic surface tensions. *J. Phys. Chem. B* **113**, 6378–6396 (2009). 19366259 10.1021/jp810292n

[81] Álvarez-Moreno, M. et al. Managing the computational chemistry big data problem: the ioChem-BD platform. *J. Chem. Inf. Model.* **55**, 95–103 (2015). 25469626 10.1021/ci500593j

