# Supplementary material for: Carbonate anions and radicals induce interfacial water ordering in CO2 electroreduction on gold
Source: Nat Chem. 2025 Nov 25;18(3):473–81. doi: 10.1038/s41557-025-01977-8 (PMC12962966; doi:10.1038/s41557-025-01977-8)
Supplement: Supplementary file 1 — Supplementary Figs. 1–20, Tables 1–3, Notes 1–5 and Discussion. [file 41557_2025_1977_MOESM1_ESM.pdf]

nature chemistry

Supplementary information

https://doi.org/10.1038/s41557-025-01977-8

Carbonate anions and radicals induce interfacial water ordering in CO_2 electroreduction on gold

In the format provided by the authors and unedited

Supplementary Information

1. **Experimental details**
2. **Stability and integrity of Au film on Si wafer**
   Supplementary Figure 1. SEM images of Au coated Si wafer before and after EC-tests
   Supplementary Figure 2. EDX mappings on the cross-section of the Au coated Si wafer before EC-tests
   Supplementary Figure 3. EDX mappings on the cross-section of the Au coated Si wafer after EC-tests
   Supplementary Figure 4. XPS data of Au-coated Si wafer before and after EC-tests
3. **ATR-SEIRAS on Au in Ar-saturated electrolyte**
   Supplementary Figure 5. *In situ* ATR-SEIRAS recorded on Au in Ar-saturated KCl electrolyte
   Supplementary Table 1. Assignments of vibrational bonds
   Note 1: Au-H bond assignment
   Note 2: Specific adsorption of carbonate.
   Supplementary Figure 6. *In situ* ATR-SEIRAS recorded on Au in Ar-saturated $KHCO_3$ electrolyte
   Supplementary Figure 7. Stability of $*CO_3^{2-}$ with applied potential (U) assessed by Computational Hydrogen Electrode (CHE) vs. by Grand-Canonical DFT (GCDFT)
   Supplementary Figure 8. On-line DEMS recorded on Au in Ar-saturated electrolyte
4. **Analysis of carbonate anion radical**
   Note 3. Discussion on the presence of $CO_3^{\bullet-}$ in water
   Supplementary Table 2. Computed parameters of $CO_3^{\bullet-}$
   Supplementary Figure 9. $CO_3^{\bullet-}$ constructions on Au (111) and Au (100)
   Supplementary Figure 10. Adsorption energies and frequencies of $CO_3^{\bullet-}$ with hydration H_2O on Au
   Supplementary Figure 11. Alignment of HOMOs of $*CO_3^{2-}$ adsorbed on (100) surfaces of different metals (M = Au, Ag, Cu, Pd, and Pt) with hydrated water LUMO.
5. **Isotopic labelling ATR-SEIRAS on Au in Ar-saturated electrolyte**
   Supplementary Figure 12. *In situ* ATR-SEIRAS recorded on Au in Ar-saturated D_2O electrolyte
   Supplementary Figure 13. *In situ* ATR-SEIRAS recorded on Au in Ar-saturated $KH^{13}CO_3$ electrolyte
   Supplementary Figure 14. Time dependent IR of the $KH^{13}CO_3$ dissolution in Ar saturated H_2O
   Supplementary Table3: Simulated and experimental IR features of possible radical ions during CO_2RR

6. **Quantitative analysis for the carbon source identification**
Supplementary Figure 15. On-line DEMS for evaluating the amount of CO_2 consumed under reduction
Supplementary Figure 16. *Operando* IR and DEMS analysis of the HCO_3^-/CO_2 equilibrium
7. **Simulated CO_2RR pathway under different applied potential**
Supplementary Figure 17. Gibbs energy profiles of CO_2 reduction to HCHO and CO
8. **Details of interfacial water effect on HER**
Note 4. Details on HER pathway
Supplementary Figure 18. Dynamic interfacial H_2O peak shift on Au in Ar- and CO_2-saturated electrolyte
Note 5. Peak fitting details
Supplementary Figure 19: Spectroscopic study of interfacial H_2O ordering in Ar-saturated electrolyte
9. **Simulated role of $CO_3^{\bullet-}$ in HER and HCHO formation**
Supplementary Figure 20. Gibbs energy profiles of $CO_3^{\bullet-}$ reduction and HER

1. Experimental details

Chemicals and Instruments. All electrochemical measurements were performed utilizing a commercial potentiostat (Biologic, Model SP-200), a reference hydrogen electrode (Mini-HydroFlex, Gaskatel GmbH) and a high-purity carbon rod (99.9995%, Sigma-Aldrich) as counter electrode. Argon and carbon dioxide gases of 99.999% purity were purchased from Air Liquide. The electrolyte was prepared using potassium bicarbonate (99.95%, Sigma-Aldrich) and ultrapure water (18.2 MΩ).

2. Stability and integrity of Au film on Si wafer

In situ ATR-SEIRAS measurements and EC-tests included cyclic voltammetry (CV) to condition and clean the Au surface in 0.5 M H_2SO_4 (from 0.3 V_{RHE} to 1.5 V_{RHE} at a scan rate of 50 mV/s), followed by linear sweep voltammetry (LSV) scans from 0.3 V_{RHE} to –1.0 V_{RHE} at a scan rate of 2 mV/s in $KHCO_3$ electrolyte. Supplementary Fig. 1 presents SEM images showing that the deposited Au remained stable after spectroscopic measurements. The Au film stability is further confirmed by EDX measurements, conducted on the cross-section of the Au-coated Si wafer before (Supplementary Fig. 2) and after spectroscopic/electrochemical measurements (Supplementary Fig. 3). In Supplementary Fig. 2, a distinct boundary between the Au film and the Si wafer substrate is visible along the red dashed line, confirming full coverage of the Si surface by the Au film. Two artificial symmetric straight lines indicate the electron beam focus area, as seen in both focused and defocused SEM images. The EDX mappings in Supplementary Fig. 3 reveal that the Au film remained stable with no Si surface exposure post-electrochemical measurements. Both SEM and EDX analyses confirm that the Au film uniformly covered the Si wafer before and after the ATR-SEIRAS measurements, including exposure to 0.1 M $KHCO_3$ electrolyte at reducing potentials. In addition, surface sensitive XPS data (Supplementary Fig. 4) confirms a pure Au surface without any detectable Si signal before and after the spectroscopic measurements. This indicates the absence of Si on the Au surface during CO_2RR and confirms that the observed IR fingerprints originate from adsorbed species (non-Si) on the Au surface.

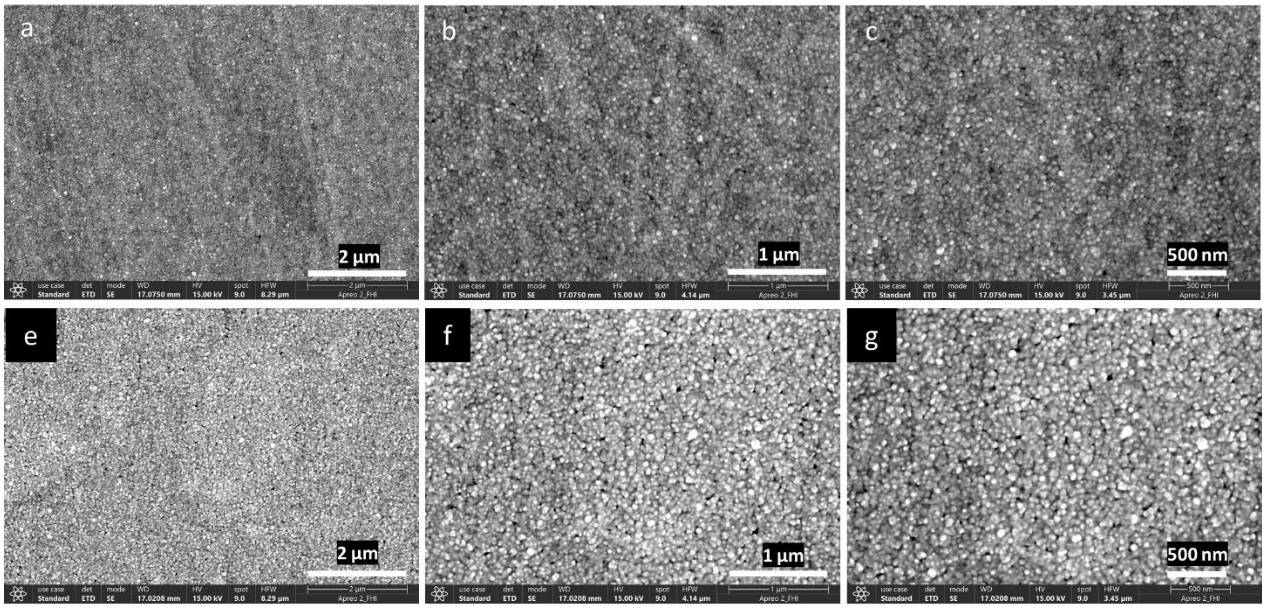

Supplementary Figure 1: SEM images of Au coated Si wafer. SEM of Au covered Si wafer before EC-tests (a, b, c) and after EC-tests (e, f, g).

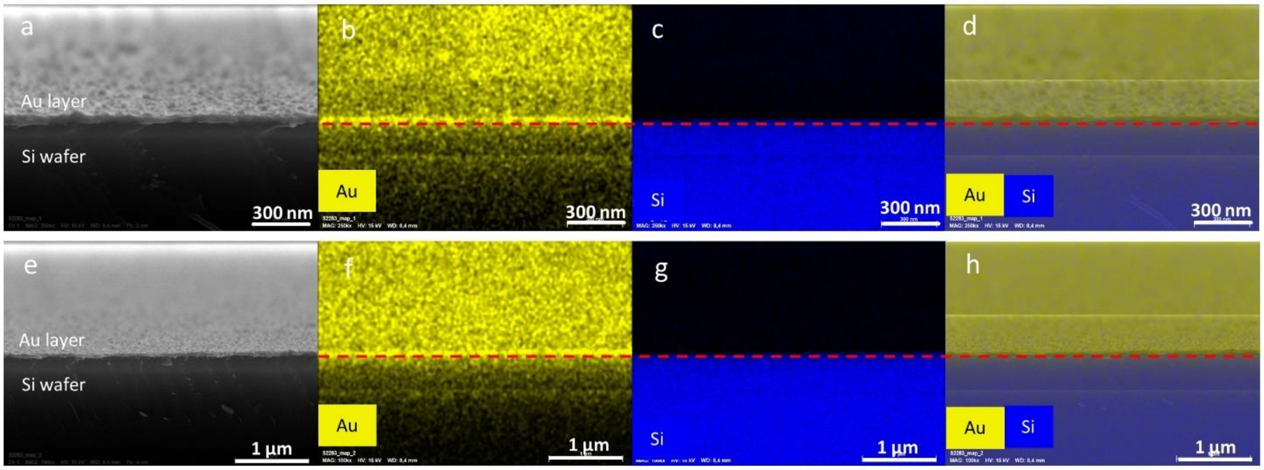

Supplementary Figure 2. EDX mappings on the cross-section of the Au coated Si wafer before EC-tests. SEM images (a, e), Au EDX mappings (b, f), Si EDX mappings (c, g) and mixed EDX mappings (d, h). Red dash line indicates the boundary between Au and Si.

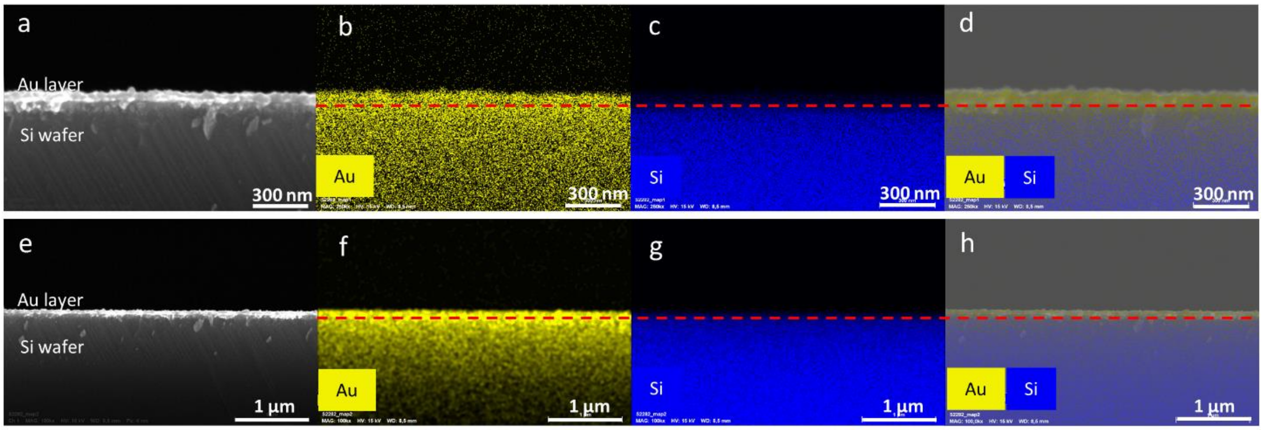

Supplementary Figure 3. EDX mappings on the cross-section of the Au coated Si wafer after EC-tests. SEM images (a, e), Au EDX mappings (b, f), Si EDX mappings (c, g) and mixed EDX mappings (d, h). Red dash line indicates the boundary between Au and Si.

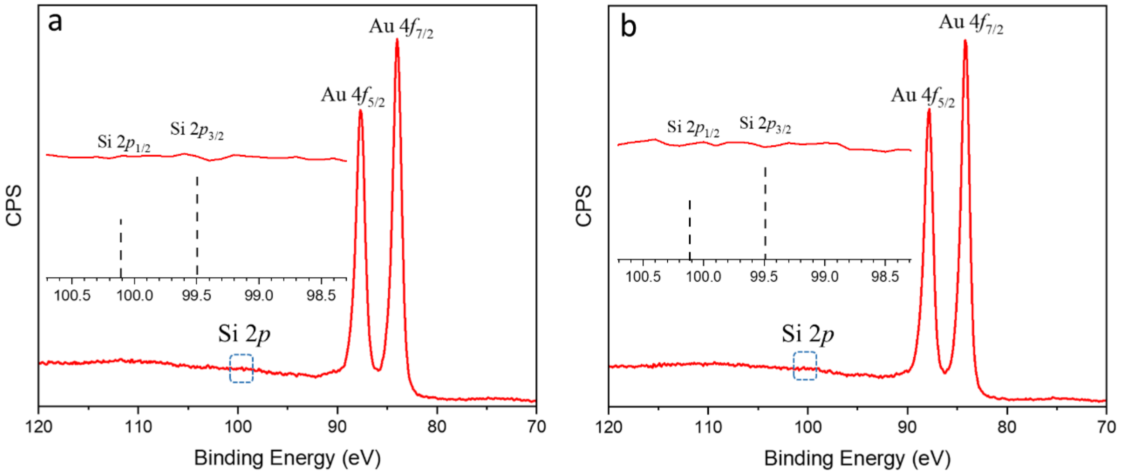

Supplementary Figure 4. XPS data of Au-coated Si wafer. (a) Au-coated Si wafer before spectroscopic measurements. (b) Au-coated Si wafer after spectroscopic measurements in glass cell. The insert diagrams show the details of Si 2p region in red box. The black dash lines represent the position of Si $2p_{1/2}$ and Si $2p_{3/2}$. The dark-yellow dash lines represent the position of Au $2f_{5/2}$ and Si $2f_{7/2}$.

3. ATR-SEIRAS on Au in Ar-saturated electrolyte

To further confirm the absence of Si substrate interference, *in situ* ATR-SEIRAS measurements were performed in Ar purified 0.1 M KCl electrolyte, excluding the presence of carbonate, bicarbonate and CO_2. Unlike in the $KHCO_3$ solution, no vibrational features at 2100 cm^{-1} or 1100 cm^{-1} were observed (Supplementary Fig. 5), indicating no detectable signals from the Si substrate. The IR data recorded in KCl align well with the cross-section SEM, EDX mapping, and surface XPS results, confirming that all detected vibrational bands originate from the Au surface.

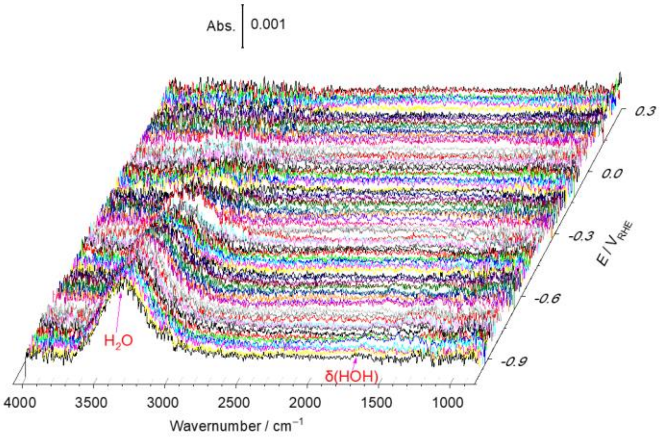

Supplementary Figure 5. *In situ* p-polarized ATR-SEIRA spectra recorded on Au surface in 0.1 M KCl electrolyte. Reference spectrum was measured at 0.3 V_{RHE}.

Supplementary Table1: Assignment of ATR-SEIRAS vibrational bonds observed in this work

Wavenumber cm^{-1}	Assignment
3600	"Dangling O-H bond" of water molecules[1-9]
3400	O-H stretching band of trihedral structure H_2O[1-9]
3200	O-H stretching band of tetrahedral structure H_2O[1-9]
2857	Overtone asymmetric C-O stretching bond of CO_3^{2-} [10-14]
2631	O-H stretching bond of HCO_3^- [12,13]
2343	Asymmetric O=C=O stretching bond of CO_2 [15,16]
2100	Co-adsorption of Au-H bond and interfacial H_2O [17-20]
1734	Stretching C=O bond of HCHO[15,21]
1672	Overtone CO_3 out of plane deformation vibration from HCO_3^- [12,13]
1650	Symmetric O-H bending bond of H_2O[7,22]
1620	Asymmetric C-O stretching bond of HCO_3^- [12-14]
1485	Symmetric CH_2 bending bond of HCHO[23]
1390	Asymmetric C-O stretching bond of hydrated CO_3^{2-} [10-12,14,24,25]
1356	Symmetric C-O stretching bond of HCO_3^- [12-14,24]
1300	C-OH bending bond of HCO_3^- [12,13,24]

1100	Symmetric C-O stretching bond of hydrated $CO_3^{\bullet-}$ [25-27]
1011	C-OH stretching bond of HCO_3^- [12,13]
846	CO_3 out of plane deformation vibration from hydrated CO_3^{2-} [10,25]

Note 1: Au-H bond assignment. SEM, EDX and XPS data confirm that the observed IR signal originate from the pure Au film due to the full covered Au film remains stable without the Si signal or other contamination before and after the spectroscopic measurements. The Volmer reaction, involving the formation of Au-H, has been identified as the rate-determining step (RDS) of the HER.[17,28,29] In Supplementary Figure 6a, a broad IR peak is observed at ~2100 cm^{-1} below –0.84 V_{RHE} in CO_2-saturated electrolyte (Fig. 1a), and within the range of –0.56 to –0.77 V_{RHE} in Ar-saturated electrolyte, respectively. Consistent with findings from previous studies based on *in situ* surface-enhanced Raman spectroscopy (SERS) and surface-enhanced infrared absorption spectroscopy (SEIRAS),[17,30] we attribute this band to an adsorbed hydrogen atom bonded to a surface Au atom (Au-H). In bicarbonate electrolyte, Fig. 3 and Supplementary Fig. 19 show that the H bonded atop the Au surface atom exhibits the same potential dependence as the observed dangling O-H bond. This indicates that hydration water with dangling O-H bonds serve as a proton donor for forming Au-H. Additionally, our spectroscopic data (Fig. 2a and Fig. 3a, 3b) and theoretical modeling (Fig. 2b and Fig. 3c-e) demonstrate that hydrated carbonate species directly lower the reaction energy of proton migration from hydration water to the Au surface atom. Linearly adsorbed CO (CO_L) on Au surfaces is expected to manifest near 2140 cm^{-1}, featuring a narrow bandwidth (FWHM, full width at half maximum) of ~23 cm^{-1}.[31] While the sum frequency of scissoring and wagging modes of H_2O typically forms a broad peak ranging from 2500 cm^{-1} to 1900 cm^{-1}, the observed peak at 2100 cm^{-1} is notably narrower thus it is assigned to the Au-H in atop configuration.

Note 2: Specific adsorption of carbonate. Within the electric double layer, anions or polar molecules can overcome electrostatic repulsion from charged surface and undergo specific adsorption (covalent bonding) and hydrogen bonding (van der Waals forces), as seen with adsorbed anions such as $HCOO^-$, CO_3^{2-}, $C_2O_4^{\bullet-}$, and $CO_2^{\bullet-}$ on electrode surfaces during CO_2RR.[15,32-38] In Fig. 1a and Supplementary Fig. 6a, p-polarized IR data indicate specifically adsorbed carbonate on the Au surface via an Au-O bond, which also forms hydrogen bonds with water molecules (Supplementary Fig. 18), suggesting the formation of a likely ordered interfacial water network at the inner Helmholtz plane (IHP). Additional simulations of hydrated carbonate with two explicit

water molecules were carried out using a Grand Canonical DFT (GCDFT) approach (Supplementary Fig. 7) to include potential and implicit solvent and cations, showing favored $*CO_3^{2-}$ adsorption energies on the Au surface at strong reductive potentials below –0.8 V_{RHE} where local cation concentration is high. This molecular and computational evidence directly confirms adsorbed carbonate on the Au surface and its role in promoting the organization of the interfacial water network."

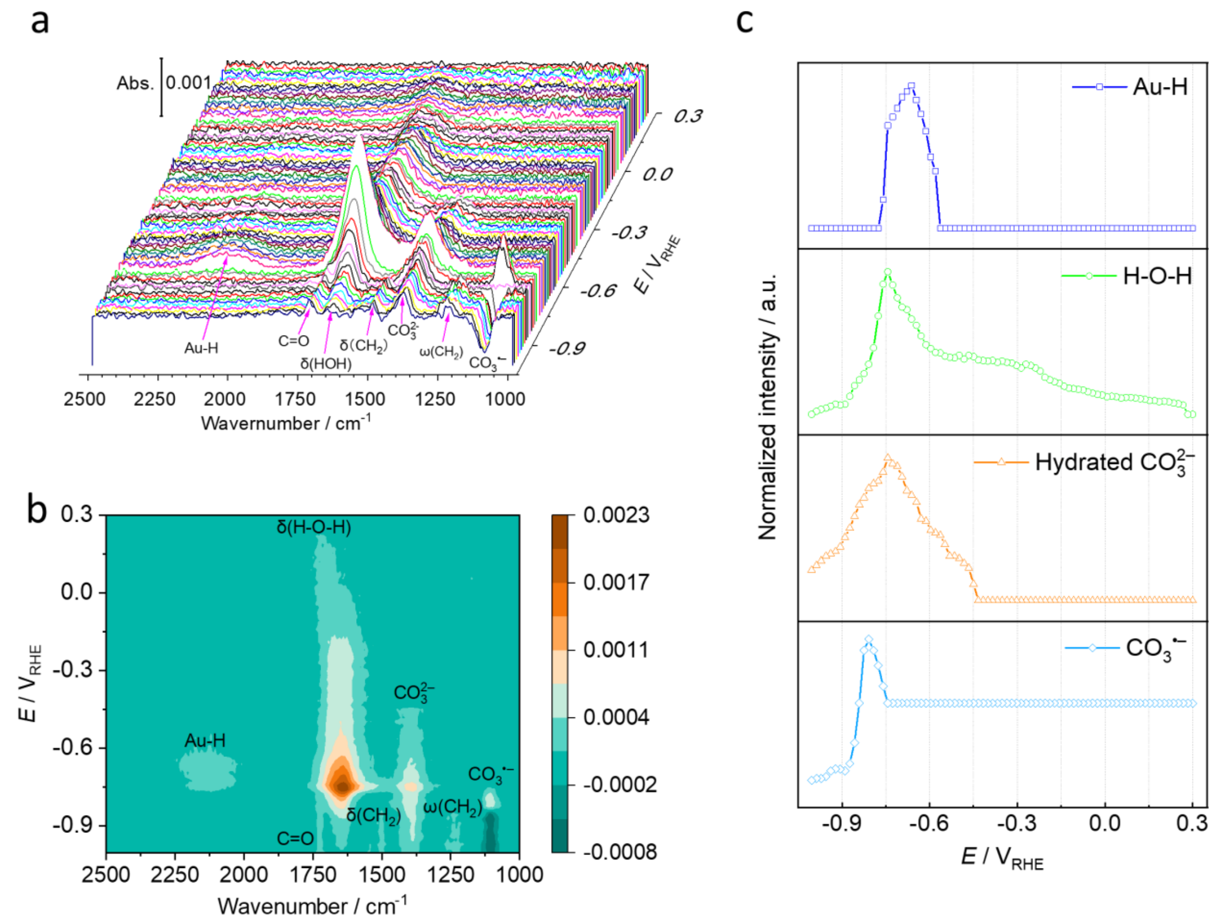

Supplementary Figure 6: *In situ* p-polarized ATR-SEIRA spectra (**a**), contour plot of interfacial spectra at different potentials **(b)**, and corresponding normalized IR band intensity recorded on polycrystalline Au in Ar-saturated 0.1 M $KHCO_3$(**c**).

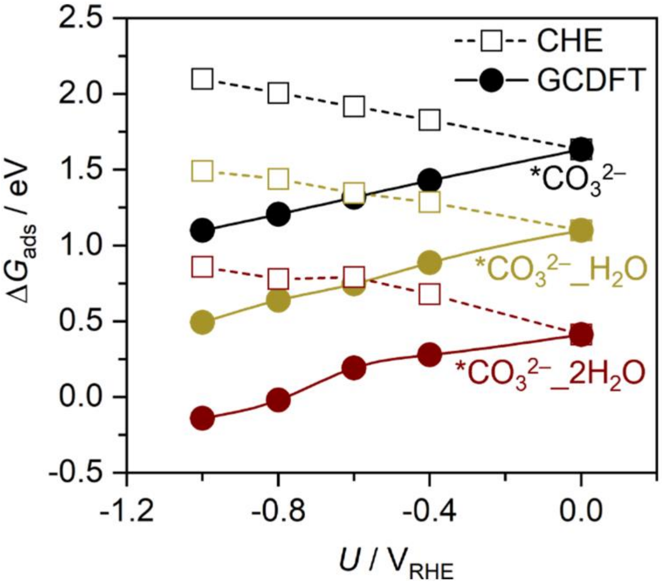

Supplementary Figure 7: Stability of *CO_3^{2-} with applied potential (*U*) assessed by Computational Hydrogen Electrode (CHE) vs. by Grand-Canonical DFT (GCDFT). Gibbs free energies of carbonate adsorption (ΔG_{ads}) on Au(100) vs. the applied potential (U), which is included by means of CHE corrections (dashed lines) and by GCDFT approach (solid lines). Simulations including 0, 1, and 2 implicit H_2O molecules are represented in black, yellow, and red, respectively.

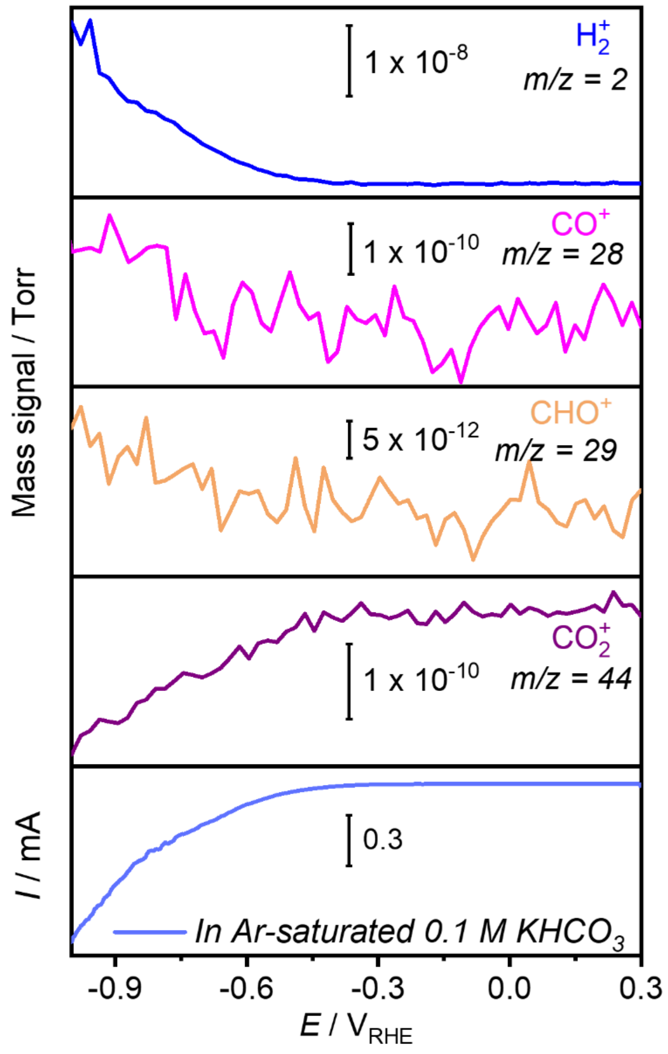

Supplementary Figure 8: Mass signal of H_2 (m/z = 2), CO (m/z = 28), CHO (m/z = 29, aldehyde group) and CO_2 (m/z = 44) and the Faradaic current for polycrystalline Au in Ar-saturated 0.1 M $KHCO_3$ as measured in the negatively going potential sweep from 0.3 V_{RHE} to −1.0 V_{RHE} at 2 mVs^{-1}.

4. Carbonate anion radical

Note 3: Presence of $CO_3^{\bullet-}$ in water. Carbonate radical anions ($CO_3^{\bullet-}$) have been widely studied over the past decade due to their presence in natural water and biological systems.[39-43] Notably, the steady-state concentration of $CO_3^{\bullet-}$ in natural water is approximately two orders of magnitude higher than that of $^{\bullet}OH$.[41,44] Additionally, it has been shown that sunlight can drive the conversion of carbonate to carbonate radicals, contributing to water's self-cleaning properties.[42] In Ar-saturated electrolyte, the $CO_3^{\bullet-}$ band (Supplementary Fig. 6a) exhibits a similar potential-dependent band intensity and a slightly shifted peak compared to the IR band of carbonate (Supplementary Fig. 6b), indicating that carbonate radical ($CO_3^{\bullet-}$) formation is linked to the presence of carbonate. The $CO_3^{\bullet-}$ band (Supplementary Fig. 16a) was also observed upon adding $KHCO_3$ into Ar-purified H_2O, further confirming the formation and presence of carbonate radicals in bicarbonate solutions. These findings confirm that surface-bound carbonate radicals are in the detection regime of in situ ATR-SEIRAS spectroscopy.

The conversion of CO_3^{2-} into $CO_3^{\bullet-}$ is widely recognized across environmental science, biologic and chemistry, though its conversion mechanism remains debated.[39-43] In our study, carbonate and carbonate anion radicals appear simultaneously upon adding $KHCO_3$ in Ar-purified H_2O (Supplementary Fig. 16), suggesting that $CO_3^{\bullet-}$ forms via charge transfer from carbonate to the hydration water. Moreover, in Ar purified $KHCO_3$ electrolyte (Supplementary Fig. 6b), the similar band intensity and slight shift in peak position relative to the carbonate IR band indicate that $CO_3^{\bullet-}$ originates carbonate. This charge transfer is more likely under cathodic potential on Au, as the CO_3^{2-} HOMO is shifted to higher energy levels than the LUMO of hydrated H_2O, enabling electron transfer (Fig. 1e).

Supplementary Table2: Magnetization values and Bader charges q_B (in $|e^-|$) for each $CO_3^{\bullet-}$ atoms of the computed structures fully relaxed, with fixed C-O$^{\bullet}$ bond, and with all bonds fixed. Bond distances and computed frequencies are depicted in Supplementary Fig. 9.

	Au(100)			Au(111)		
	relaxed	C-O$^{\bullet}$ fix	all fix	relaxed	C-O$^{\bullet}$ fix	all fix
C	–0.01	–0.02	–0.02	0.00	–0.02	–0.02
O (1)	0.17	0.35	0.40	–0.00	0.34	0.39
O (2)	0.06	0.07	0.08	–0.00	0.07	0.07
O (3)	0.06	0.07	0.08	–0.00	0.07	0.08
q_B	–0.84	–0.81	–0.80	–0.83	–0.77	–0.74

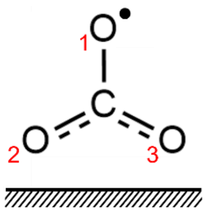

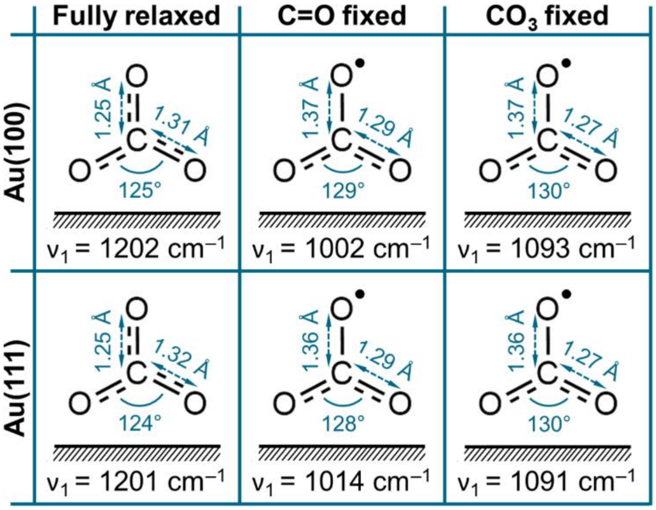

Supplementary Figure 9: The computed carbonate radical ($CO_3^{\bullet-}$) constructions on Au(111) and Au(100).

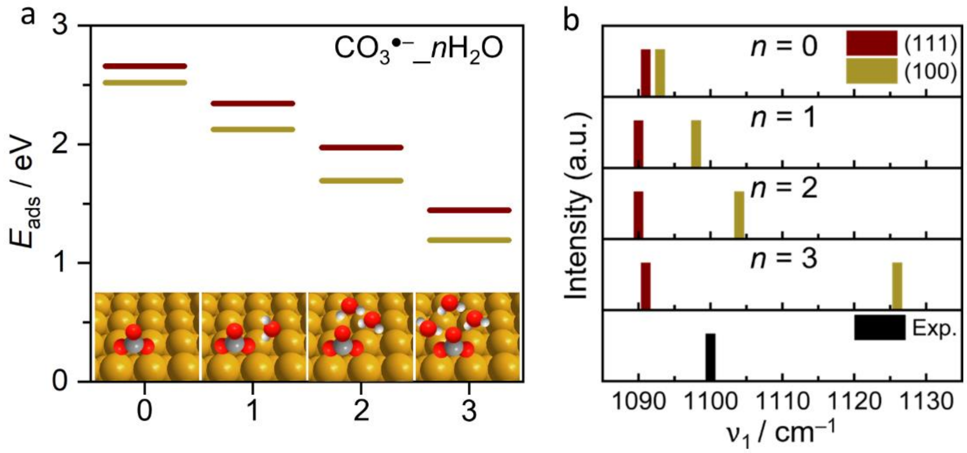

Supplementary Figure 10: Adsorption energies (a) and ν_1 frequencies (b) of the computed carbonate radical ($CO_3^{\bullet-}$) with hydration of $n$$H_2O$ molecules (n = 0, 1, 2, and 3) on Au surfaces (100) and (111). Panels show the structures with Au (yellow), C (gray), O (red), and H (white) atoms.

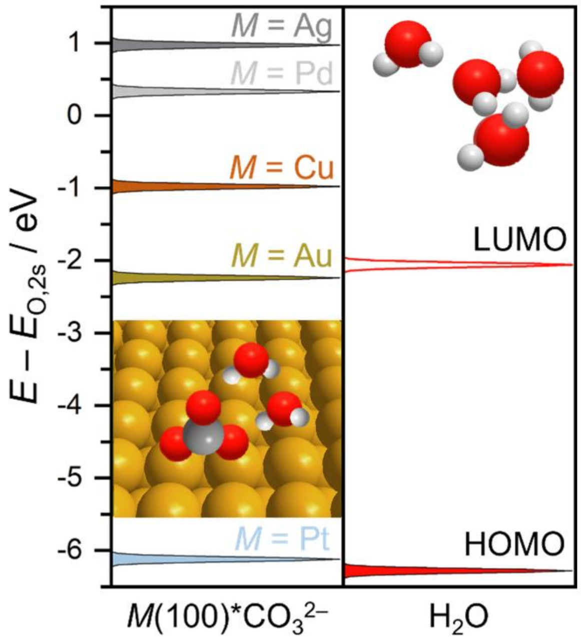

Supplementary Figure 11: Alignment of HOMOs of $*CO_3^{2-}$ adsorbed on (100) surfaces of different metals (*M* = Au, Ag, Cu, Pd, and Pt) with hydrated water LUMO. Panel at the left represents the adsorbed carbonate on Au(100), the one with the closest HOMO to hydrated H_2O (computed as a gas phase H_2O molecule surrounded by 3 explicit waters, panel at the right) LUMO. Au, Ag, Cu, Pd, and Pt energy levels are depicted in yellow, dark grey, brown, gray, and blue, respectively. Au, C, O, and H atoms in panels are depicted in yellow, grey, red, and white, respectively.

5. Isotopic labelling ATR-SEIRAS on Au in Ar-saturated electrolyte

D_2O labeling was used to further confirm the assignment of the ~2100 cm^{-1} vibrational band. As shown in Supplementary Fig. 12a, the absence of the ~2100 cm^{-1} band in Ar-purged D_2O-based $KHCO_3$ electrolyte provides additional evidence that this vibrational feature originates from the Au-hydrogen stretching mode. The O-D bending band of water appears near 1270 cm^{-1}, alongside a band at ~ 1460 cm^{-1} attributed to the adsorbed deuterium on Au surface. The isotopic shift of the Au-H bond (Supplementary Fig. 12b) aligns well with harmonic oscillator predictions, supporting its assignment to surface-bound hydrogen. The spectral overlap between the O-D bending mode and adsorbed deuterium indicates their co-adsorption structure and strong interactions on Au electrode. Such hydrogen-water interactions have previously been elucidated under ultra-high vacuum conditions,[45] and similar co-adsorption configurations were reported on Pt (100) single crystal.[19] These isotopic IR results further support that the ~2100 cm^{-1} band in H_2O-based electrolyte originates from the co-adsorption hydrogen and water.

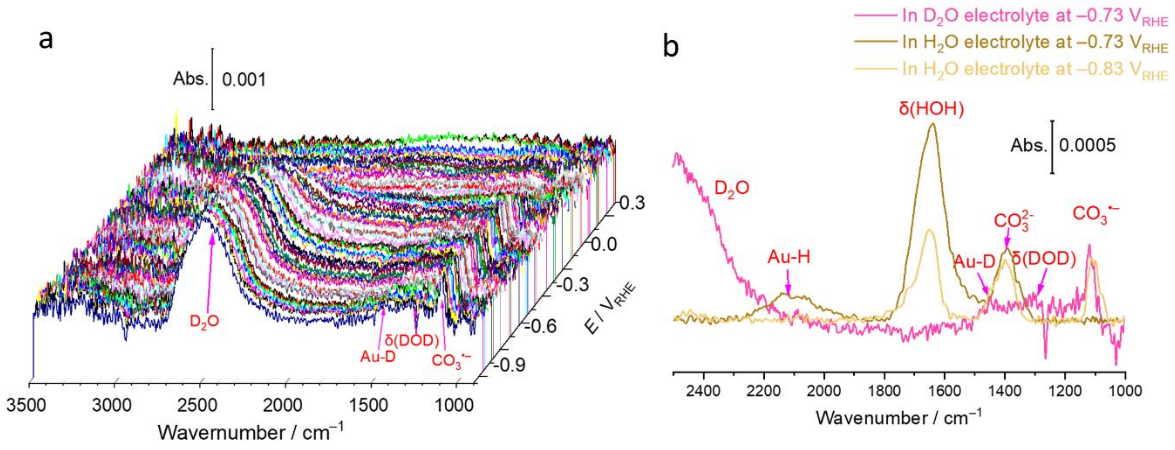

Supplementary Figure 12: (a) In situ p-polarized ATR-SEIRAS spectra recorded on the Au surface in an Ar-saturated D_2O $KHCO_3$ electrolyte, (b) single spectrum recorded in Ar purified D_2O electrolyte. Reference spectrum was recorded at 0.3 V_{RHE}.

In a $KH^{13}CO_3$ solution, a stronger vibrational band appears at ~2100 cm^{-1} compared to $KHCO_3$(Supplementary Fig. 6b), shifting to ~2150 cm^{-1} with broadened FWHM, from –0.32 V_{RHE} to –0.80 V_{RHE} (Supplementary Fig. 13b). This shift cannot be attributed to the Stark effect, as the expected Stark shift occurs in opposite direction (toward lower frequencies) under applied negative potential. The band located at ~2150 cm^{-1} (spanning 2500 cm^{-1} to 1800 cm^{-1}) corresponds to the sum frequency of H_2O scissoring and wagging modes, while the ~2100 cm^{-1} band (2300-1900 cm^{-}

^{1}) shows the signature of adsorbed hydrogen on the noble metal electrodes, particularly in neutral and basic solutions[18-20,45-47]. The broad Au-H band indicates variations in coverage, bonding configuration and interactions with interfacial water. The observed transition from adsorbed hydrogen to interfacial water (Supplementary Fig. 13b) indicates their co-existence and competitive balance on the Au electrode under reducing conditions. Inherently, the ~2100 cm^{-1} peak relates to co-adsorption of hydrogen and water on Au. Moreover, the potential-dependent IR intensity of adsorbed hydrogen (Supplementary Fig. 13a-b) correlates well with the intensity of CO_3^{2-}, reinforcing the crucial role of CO_3^{2-} in inducing an ordered water network that facilitates Au-H formation.

In symmetric stretching, atomic motion is highly coordinated. As shown in Fig. 1b, the central carbon atom remains nearly stationary, while the oxygen atoms move symmetrically. The reduced mass of ν_s(C-O) is primarily determined by the oxygen, so substituting the central carbon with isotope ^{13}C has minimal impact (< 3%) on the reduced mass and does not significantly alter the symmetry of the vibrational mode. Consequently, the vibrational frequency remains largely unchanged, as the force constant is unaffected by isotopic mass. These negligible changes are insufficient to cause a detectable shift in the of symmetric C-O vibrational frequency of $CO_3^{\bullet-}$. This is confirmed by operando IR measurements with ^{13}C labeling (Supplementary Fig. 13c), which show no isotope shifts at ~1100 cm^{-1}, supporting the $CO_3^{\bullet-}$ assignment. In contrast, the asymmetric C-O stretching mode of $CO_3^{2-}$where the oxygen atoms move out-of-phase, exhibits a significant shift from 1396 cm^{-1} to 1352 cm^{-1}. Here, the central carbon atom's motion is more pronounced, making the vibrational frequency more sensitive to isotope substitution.

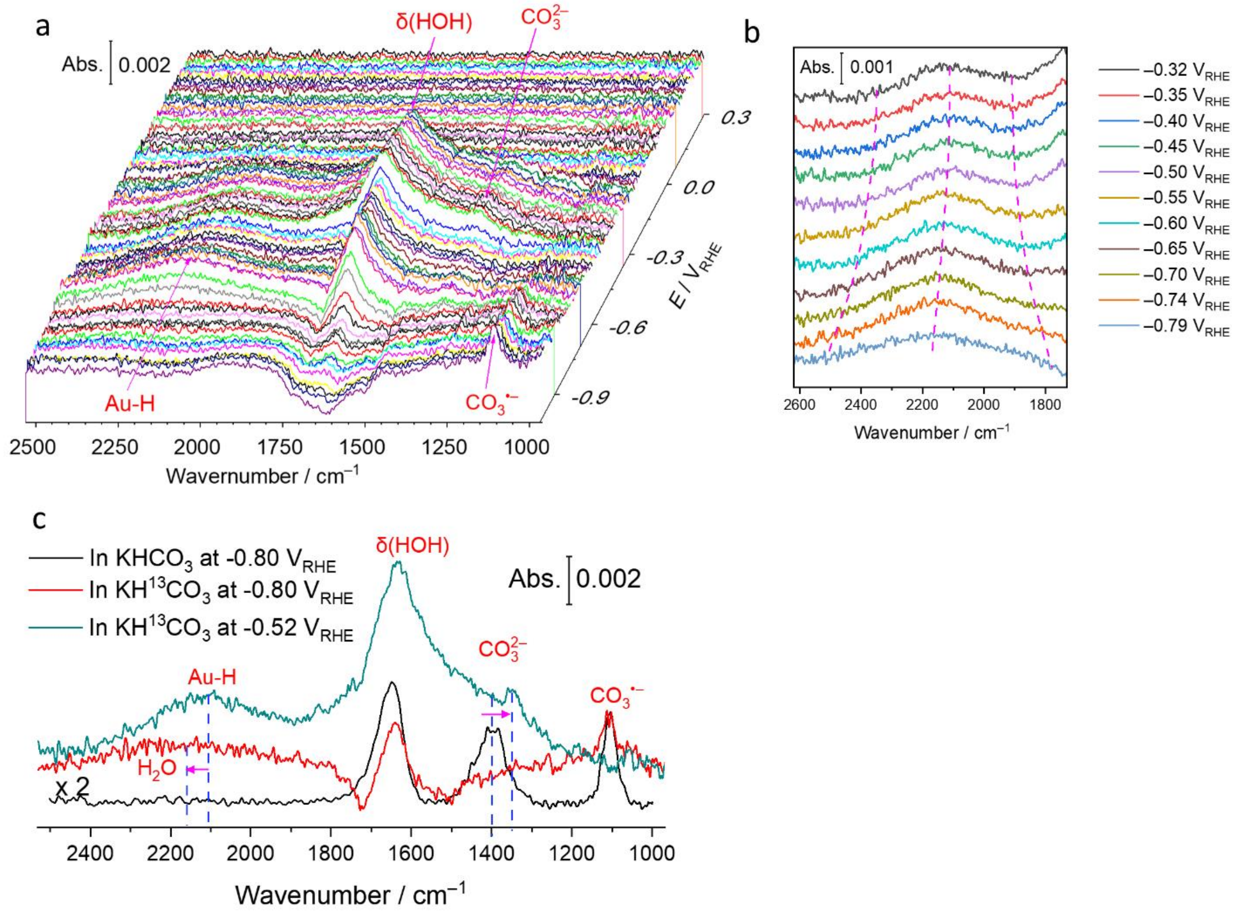

Supplementary Figure 13: (a) *In situ* p-polarized ATR-SEIRAS spectra recorded on Au surface in Ar-purified $KH^{13}CO_3$ electrolyte, (b) The potential dependent of vibration bands in the region from 2600 cm^{-1} to 1750 cm^{-1}, (c) single spectrum recorded in Ar purified $KHCO_3$, $KH^{13}CO^3$ electrolyte under reduction. Reference spectrum was recorded at 0.3 V_{RHE}.

The vibrational frequency at ~1100 cm^{-1} appeared several minutes after $KHCO_3$ was added to the Ar-purified H_2O (Supplementary Fig. 16), indicating that these vibrational features originates from the decomposition of $KHCO_3$ or its derivatives. A similar evolution of all fingerprints was observed when $KHCO_3$ was replaced with $KH^{13}CO_3$ (Supplementary Fig. 14), including the transient appearance of $CO_3^{\bullet-}$ at ~1100 cm^{-1}. Like $CO_3^{\bullet-}$, the symmetric C-O stretching mode of HCO_3^- shows no detectable isotope shift due to its symmetric bonding and stretching configuration (Supplementary Fig. 14b). In contrast, the asymmetric stretching ν_{as}(O=C=O) and wagging ω(C-O) modes of CO_3^{2-} and HCO_3^- (overtone) exhibit significant isotope shifts, as these vibrational modes are sensitive to isotope substitution.

The possible carbon-based radical ions are summarized in Supplementary Table3. $CO_2^{\bullet-}$ has been widely studied as the first reaction intermediate of CO_2RR,[24,29,48-51] with vibrational bands recorded at ~1559 cm^{-1} (ν_{as}), ~1400 cm^{-1} (ν_s) on Ag and Cu surfaces via *in situ* ATR-FTIR.[15,35] Compared to $CO_2^{\bullet-}$, the more stable dimer radical anion $C_2O_4^{\bullet-}$ has been reported as mechanistically relevant in CO_2RR,[52-54] with its fingerprint of $C_2O_4^{\bullet-}$ identified at ~1627 cm^{-1} via ATR-FTIR.[14,33] Our simulated frequencies for $CO_2^{\bullet-}$ (1643 and 1465 cm^{-1} for ν_{as} and ν_{as}, respectively) and $C_2O_4^{\bullet-}$ (1608 cm^{-1}) align well with these experimental observations.

Thus, the vibrational band at ~1100 cm^{-1} is unrelated to $CO_2^{\bullet-}$ (~1400, ~1559 cm^{-1}) and $C_2O_4^{\bullet-}$ (~1627 cm^{-1}). Instead, carbonate radical anions ($CO_3^{\bullet-}$) have been reported at ~1100 cm^{-1} using spectroscopic techniques complementary to IR spectroscopy and simulations.[25,27,55,56] Notably, the conversion of CO_3^{2-} into $CO_3^{\bullet-}$ has been shown to build a significant steady-state concentration of carbonate radical anions in natural water.[41,57] Therefore, the IR peaks near 1100 cm^{-1} observed during CO_2RR are attributed to carbonate radicals originating from carbonate anions, in excellent agreement with our simulated $CO_3^{\bullet-}$ fingerprint (Supplementary Fig. 9 and S10) and ^{13}C-labeled IR results (Supplementary Fig. 13 and Supplementary Fig. 14).

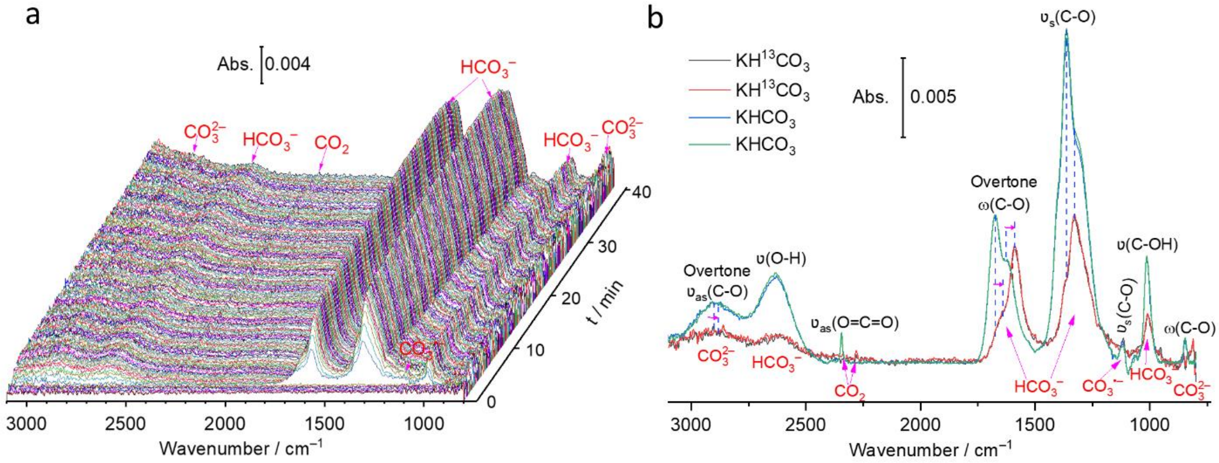

Supplementary Figure 14. (a) Time-dependent ATR-SEIRAS (S-polarization). (b) Adjacent single spectrum recorded the $KHCO_3$ and $KH^{13}CO_3$ dissolution in Ar-saturated water. Reference spectrum was recorded in Ar-saturated water.

Supplementary Table 3: Simulated and experimental IR features of possible radical ions during CO_2RR

Derived carbon-based radicals	Experimental IR locations / cm^{-1}	Simulated IR positions / cm^{-1}
$CO_2^{\bullet-}$	~1559 (υ_{as}), ~1400 (υ_s) on Cu, Ag[15,35]	~ 1643 (υ_{as}), ~ 1465 (υ_s)
$C_2O_4^{\bullet-}$	~1627 on Cu[14,33]	~ 1608
$CO_3^{\bullet-}$	~1100 on Au (This work)	~1100 on Au

6. Quantitative DEMS analysis: Carbon source of CO_2RR

Quantitative DEMS measurements were carried out to investigate the effects of mass transfer. First, the process of CO_2 bubbling in Ar-purified 0.1 M $KHCO_3$ was monitored using online DEMS, as shown in Supplementary Fig. 15. The CO_2 saturation concentration in 0.1 M $KHCO_3$ was calculated by the following equation:

$$c_S^* \, (CO_2) = c_S(CO_2) * \frac{MS_S^*(CO_2)}{MS_S(CO_2)} \qquad \text{(Eq. S1)}$$

where c_s^* *(CO_2)* and MS_s^* *(CO_2)* represent the CO_2–saturated concentration and its corresponding mass signal intensity in 0.1 M $KHCO_3$, and c_s *(CO_2)* and MS_s *(CO_2)* denote the CO_2–saturated concentration and its corresponding mass signal intensity in H_2O. The DEMS measurements of CO_2 bubbling into Ar-purified H_2O were performed under the same conditions and employed as the calibration experiment. It is known that the saturation concentration of CO_2 is approximately 34 mmol/L in water at room temperature and normal pressure. According to Eq. S1, the CO_2 concentration was determined to be 28.22 mmol / L in 0.1 M $KHCO_3$. Based on the DEMS data from the LSV shown in Fig. 1d, the actual amount of CO_2 consumption at −0.65 V_{RHE} and −1.0 V_{RHE} was calculated as 0.65 and 3.15 mmol / L in CO_2-saturated electrolyte, respectively. These quantitative findings indicate that there are no CO_2 mass transfer limitations under CO_2RR conditions, as the local CO_2 concentration is significantly higher than the amount of CO_2 consumption on the Au surface.

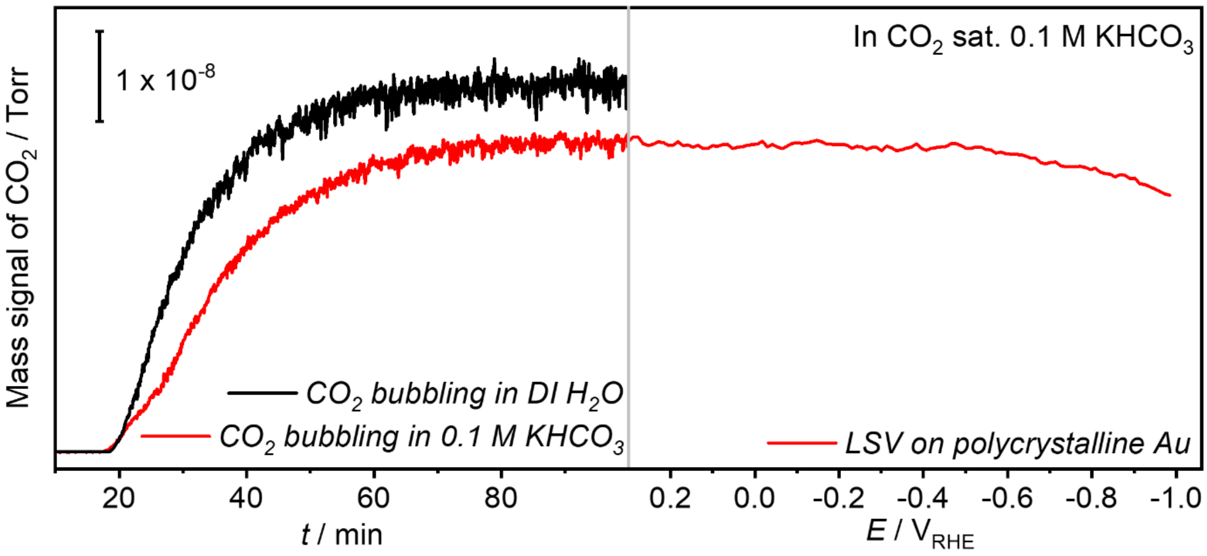

Supplementary Figure 15: Mass signal of CO_2 (m/z = 44) recorded under CO_2 bubbling in Ar-purified H_2O, Ar-purified 0.1 M $KHCO_3$, and LSV test on polycrystalline Au.

Supplementary Fig. 16a shows the real time s-polarized ATR-SEIRA spectra capturing the process of potassium bicarbonate dissolution in Ar-saturated H_2O. Initially, no bands were detected until the addition of $KHCO_3$ into the Ar-saturated H_2O. The observation of the asymmetric stretching C=O band of CO_2 at ~2343 cm^{-1} provided direct evidence for the equilibrium between bicarbonate and CO_2. Additionally, strong IR peaks observed at ~2631 cm^{-1}, ~1672 cm^{-1}, ~1620 cm^{-1}, ~1356 cm^{-1}, ~1300 cm^{-1} and ~1011 cm^{-1} were assigned to the vibrational bonds of HCO_3^-. The weak bands observed near 2857 cm^{-1} and 846 cm^{-1} originate from CO_3^{2-}. The previously reported Raman spectrum of carbonate during the dissolution of $CsHCO_3$ further supports the assignment of CO_3^{2-}.[13] Interestingly, as shown in Supplementary Fig. 16b, a very weak band at ~1100 cm^{-1} was observed several minutes after the addition of $KHCO_3$ into the Ar-purified H_2O. Its position closely resembles the vibrational frequency of the C=O bond in the carbonate anion radical ($CO_3^{\bullet-}$), suggesting possible charge transfer from carbonate to the water molecules in the first hydration sphere.[25] Based on the observed vibrational bonds from HCO_3^-, CO_2 and CO_3^{2-}, an equilibrium of bicarbonate is proposed as following:

$$2HCO_3^- \leftrightarrow H_2O + CO_2 + CO_3^{2-} \quad \text{(Eq. S2)}$$

In Supplementary Fig. 16b, it is evident that all IR bands corresponding to CO_2, HCO_3^-, and CO_3^{2-} emerge immediately upon the addition of potassium bicarbonate to Ar-saturated water. For semi-quantitative analysis, the stretch vibrational bond of C=O at ~1011 cm^{-1} is selected due to its

independence from other coupled C=O vibrational bonds. As shown in Supplementary Fig. 16c, both CO_2 and bicarbonate are observed synchronously with the same band intensity evolution, indicating their direct interrelationship. During the initial few minutes, the increasing trend of normalized band intensity for CO_2 and HCO_3^- is attributed to the dissolution process of $KHCO_3$ and mass transfer effects. In Supplementary Fig. 16d, the DEMS data show the detection of CO_2 species, as solid $KHCO_3$ is added to Ar-saturated H_2O, further supporting the rapid equilibrium between HCO_3^- and CO_2.

Previous reports have suggested that dissolved CO_2 originating from HCO_3^- serves as the primary reactant during CO_2RR, yet quantitative evidence to support this is lacking. Here, we conducted a quantitative analysis to determine the actual amount of CO_2 generated from HCO_3^- decomposition under operation conditions. The quantity of CO_2 derived from HCO_3^- in Ar-saturated $KHCO_3$ electrolyte was calculated using the following equation:

$$c\ (CO_2) = c_s(CO_2) * \frac{MS(CO_2)}{MS_S(CO_2)} \qquad \text{(Eq. S3)}$$

In Eq. S3, *c (CO₂)* represents the concentration of CO_2 in Ar-saturated $KHCO_3$ electrolyte, $c_s\ (CO_2)$ denotes the saturated concentration of CO_2 in H_2O (34 mmol/L), $MS\ (CO_2)$ indicates the intensity of the CO_2 mass signal in Ar-saturated electrolyte, and $MS_s\ (CO_2)$ represents the intensity of the CO_2 mass signal in CO_2-saturated H_2O. The concentration of CO_2 derived from HCO_3^- was determined to be 0.23 mmol/L in Ar-purified electrolyte, significantly lower than the CO_2 consumption observed during CO_2RR at −0.6 V_{RHE} (0.65 mmol/L) and −1.0 V_{RHE} (3.15 mmol/L) in the CO_2-saturated electrolyte. In CO_2-saturated electrolyte, the actual amount of CO_2 originating from HCO_3^- is expected to be lower and can be calculated using the chemical equilibrium constant of this HCO_3^-/CO_2 equilibrium:

$$K = \frac{c(CO_2)*c(CO_3^{2-})}{c(HCO_3^-)^2} \qquad \text{(Eq. S4)}$$

Here, K represents the chemical constant of the HCO_3^-/CO_2 equilibrium, $c\ (CO_3^{2-})$ is the concentration of CO_3^{2-}, which is equivalent to the concentration of CO_2 ($c\ (CO_2)$), $c\ (HCO_3^-)$ denotes the actual concentration of HCO_3^-, calculated using the equation: $c\ (HCO_3^-) = 0.1\ M - c\ (CO_3^{2-})$. The equilibrium constant of HCO_3^-/CO_2 was determined to be $K = 5.38 \times 10^{-6}$, and it was used to assess the real amount of dissolved CO_2 derived from HCO_3^- during CO_2RR in CO_2-saturated electrolyte. Considering the DEMS data in Fig. 1c, the quantity of dissolved CO_2 resulting from the rapid equilibrium of HCO_3^- was estimated to be 3.0 and 3.50 μmol/L at −0.65 V_{RHE} and

−1.0 V_{RHE}, respectively. These values are much lower than the CO_2 consumption level (0.65 mmol/L at −0.6 V_{RHE}, 3.15 mmol/L at −1.0 V_{RHE}) observed during CO_2RR in CO_2-saturated electrolyte, further indicating that bicarbonate is not the dominant carbon source of CO_2RR. Instead, bicarbonate functions as a CO_2 buffer to rapidly compensate for a small portion of CO_2 consumption in CO_2RR, and may serve as the main carbon source for CO_2RR only under distinct circumstances. For instance, in Ar-purified electrolyte, Supplementary Fig. 8 displays DEMS plots illustrating CO_2 consumption during LSV measurement, indicating that bicarbonate anions likely serve as the carbon source for CO_2RR through the rapid equilibrium of HCO_3^-/CO_2. If dissolved CO_2 were the only carbon source of CO_2RR, the carbon-containing products would be proportional to the amount of CO_2 consumption. However, as shown in Supplementary Fig. 8, the intensities of CO and formaldehyde were approximately half of those produced in CO_2-saturated electrolyte with less than one-tenth of the CO_2 consumption. These findings prompt a reconsideration of the carbon source of CO_2RR.

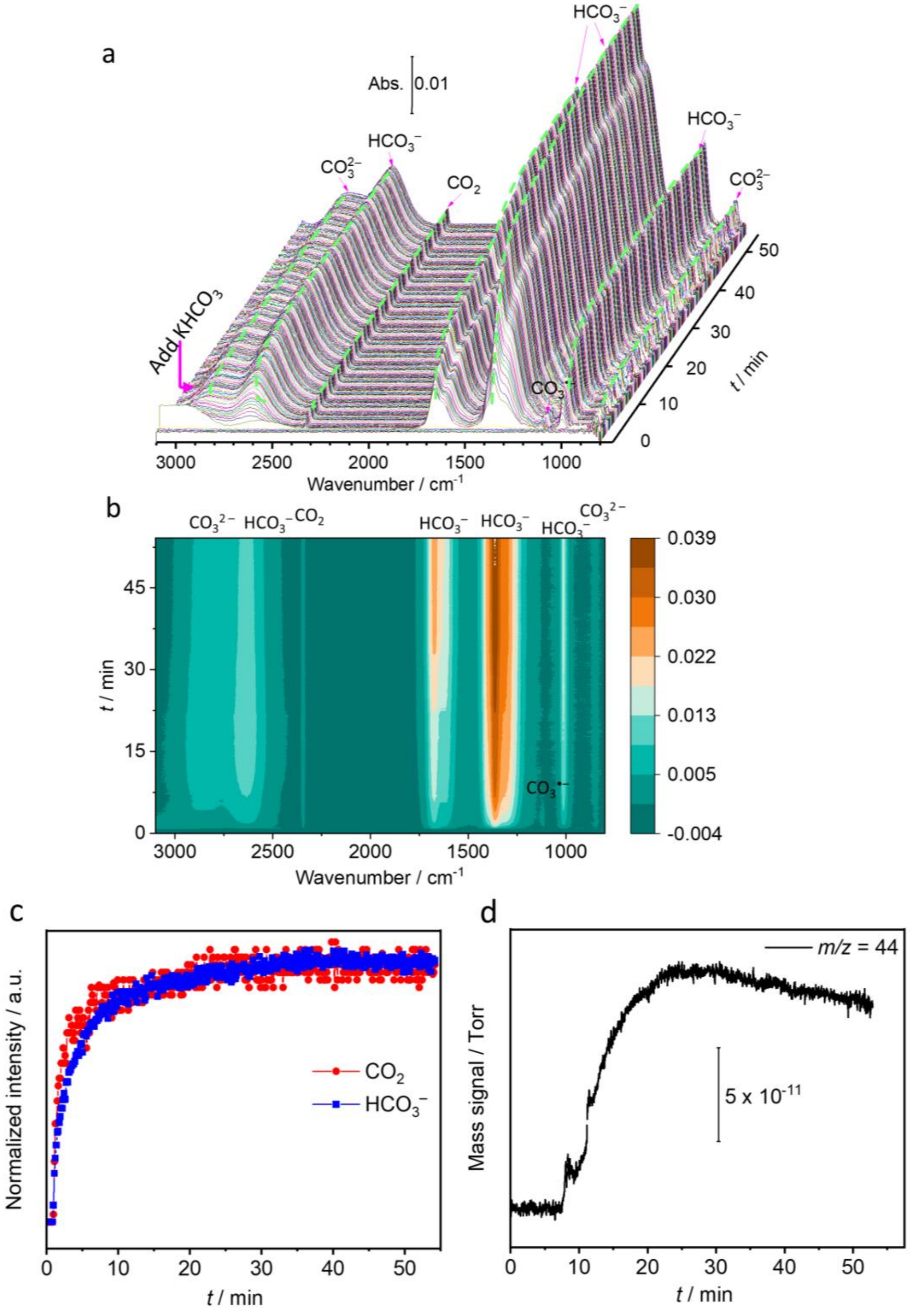

Supplementary Figure 16: (a) Time-dependent ATR-SEIRAS (S-polarization), (b) contour plot of spectra and (c) normal integrated band intensities of CO_2 and HCO_3^- recorded on polycrystalline Au film during the addition of the potassium bicarbonate in Ar-saturated water, (d) real-time mass signal of CO_2 (*m/z* = 44) recorded during the $KHCO_3$ dissolution in Ar-saturated water. Reference spectrum was recorded in Ar-saturated water.

7. Simulated CO_2RR pathway under different applied potential

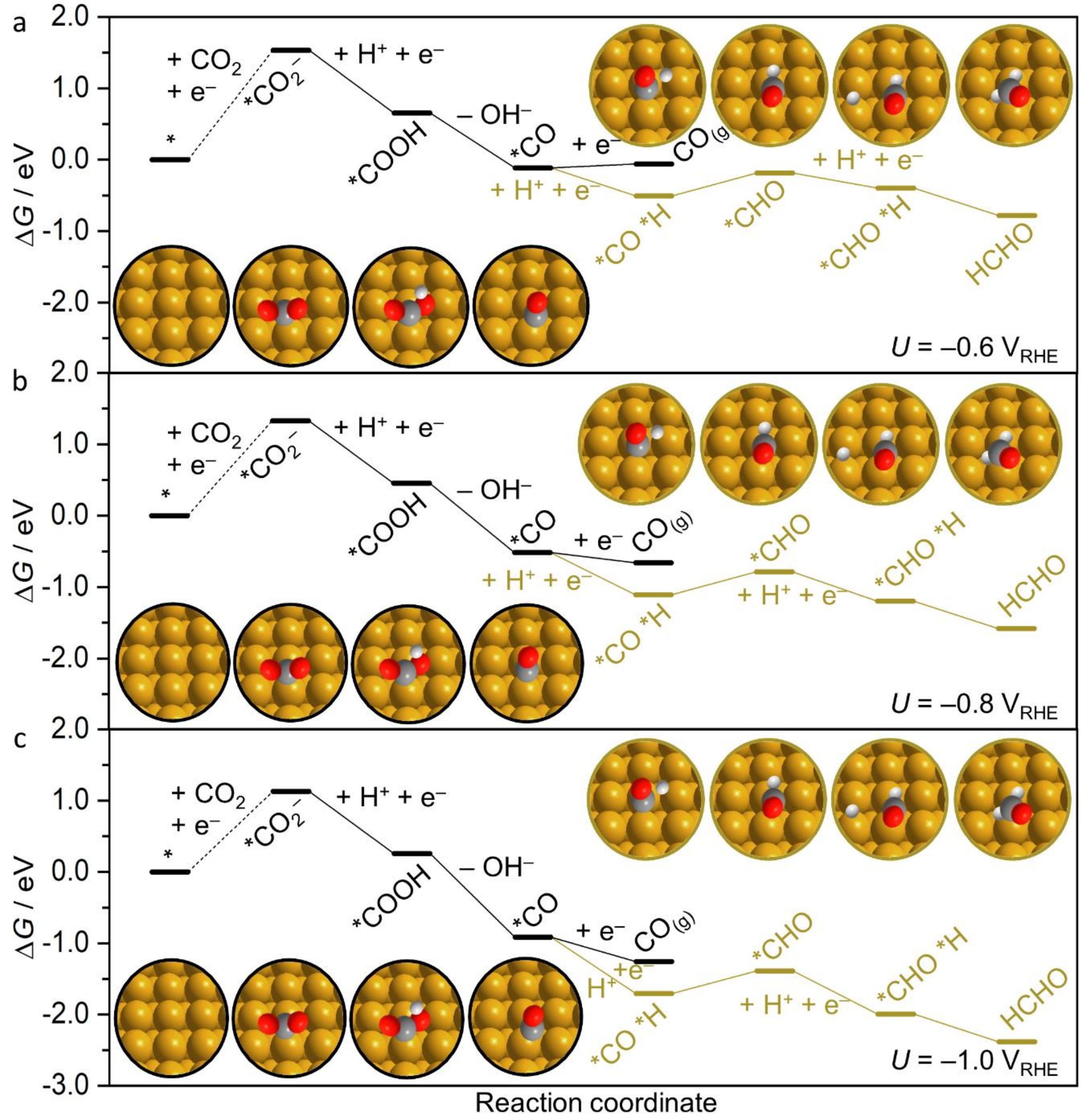

Supplementary Figure 17: Gibbs energy profiles of CO_2 reduction to HCHO (yellow) and CO (black) on Au(100) at different potentials –0.6 (a), –0.8 (b), and –1.0 (c) V_{RHE}. Reaction intermediates are depicted with Au (yellow), C (gray), O (red), and H (white) atoms.

8. Interfacial water effect on HER

Note 4: HER pathway discussion. According to the D_2O labelling measurements, the HER pathway on Au during CO_2RR in $KHCO_3$ electrolyte can be described by equations S5, S6, and S7. Due to the equilibrium reaction of HCO_3^- (Eq. S2), minor H_2O could possibly also originate from HCO_3^- in the D_2O-$KHCO_3$ electrolyte. This H_2O may participate in the Volmer step, forming an Au-H bond, followed by the Heyrovsky step, where Au-H may react with H_2O and HCO_3^- to produce H_2. However, stronger hydrogen bonds between CO_3^{2-} and D_2O compared to those in H_2O suggest that hydrated CO_3^{2-} with D_2O preferably forms Au-D rather than reacting with the limited free H_2O in D_2O electrolyte. This is supported by the absence of a mass signal for H_2 (m/z = 2) in the D_2O isotopic labelling DEMS measurement.

Volmer reaction: $H_2O + e^- + * \rightarrow H^* + OH^-$ (rate-limited step) (Eq. S5)

Heyrovsky reaction: $H^* + H_2O + e^- \rightarrow H_2 + * + OH^-$ (Eq. S6)

$H^* + HCO_3^- + e^- \rightarrow H_2 + * + CO_3^{2-}$ (Eq. S7)

As illustrated in Supplementary Fig. 18a and 18b, the potential-dependent intensity of the stretching H_2O band features a linear correlation with that of hydrated CO_3^{2-} in both Ar- and CO_2-saturated electrolytes. This correlation suggests that the hydration-shell water predominantly influences the formation of the interfacial water network on the Au surface. Moreover, the broadening of the H_2O band towards lower wavenumbers with increasing intensity of hydrated CO_3^{2-} under bias potential indicates a significant impact on the orientation of the interfacial water by hydrated CO_3^{2-}. The red shift of the interfacial water intensity implies a charge transfer (CT) from carbonate to hydration water and the formation of stronger and shorter hydrogen bonds in the presence of hydrated CO_3^{2-} .[25] These CT processes and shorter hydrogen bonds promote both the electron transfer rate and proton transfer rate in the CO_3^{2-}-induced well-ordered interfacial water network, leading to enhanced proton-coupled electron reactions.[58]

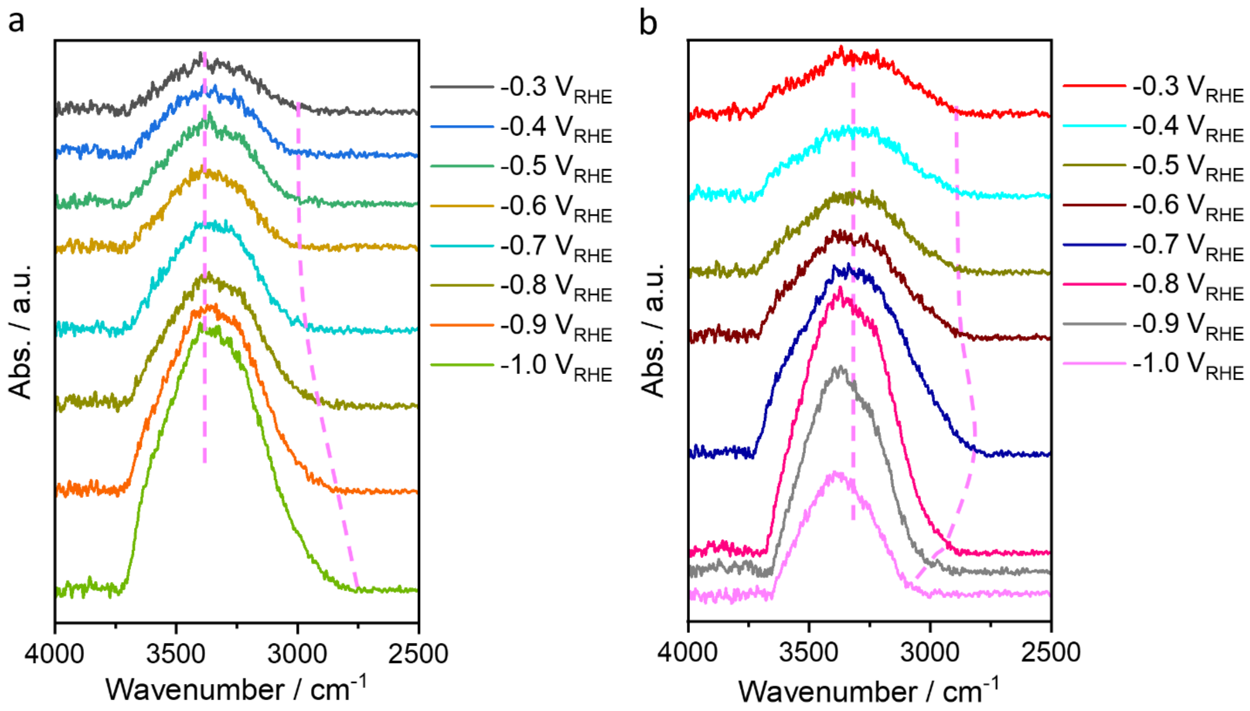

Supplementary Figure 18: *In situ* ATR-SEIRA spectra with p-polarization for a polycrystalline Au in CO_2-saturated (a) and Ar-saturated (b) 0.1 M $KHCO_3$. Reference spectrum was taken at 0.3 V_{RHE}.

Note 5: Interfacial H_2O peak fitting. The band intensity fitting procedure comprises three steps: selecting the initial profiles (line shapes and baseline handling), choice of initial parameters (width, height, location), and minimization. In this work, the peak fitting was carried out through Origin 2021b with Gaussian profile for all peaks. Prior spectral fitting, the linear baseline correction was performed to minimize the residual. The initial locations of O-H vibrations were determined as 3200 cm^{-1}, 3400 cm^{-1} and 3600 cm^{-1}, referring to the tetrahedral structure of water (Peak 1), trihedral structure of water (Peak 2), and dangling O-H bonds (Peak 3). At last, multiple iterations were performed to minimize the difference between the model and the actual data until converging. Supplementary Fig. 19 shows a decrease in interfacial water ordering below –0.7 V_{RHE}, along with a reduction in the intensity of hydrated CO_3^{2-}, providing an additional view to the predominant electrostatic cation effect (increased interfacial water ordering during cation accumulation at negative potential sweep) reported in alkaline electrolytes. This indicates that adsorbed carbonate and its hydration water at the IHP play a critical role in facilitating proton and electron transfer to the electrode surface, due to the strong connectivity of the CO_3^{2-}-induced ordered interfacial water network. Theoretical data (Fig. 2b) further support these spectroscopic findings, showing that the

presence of K^+ has minimal impact on lowering the Au-H formation energy from free water, compared to the CO_3^{2-}-induced interfacial water layer.

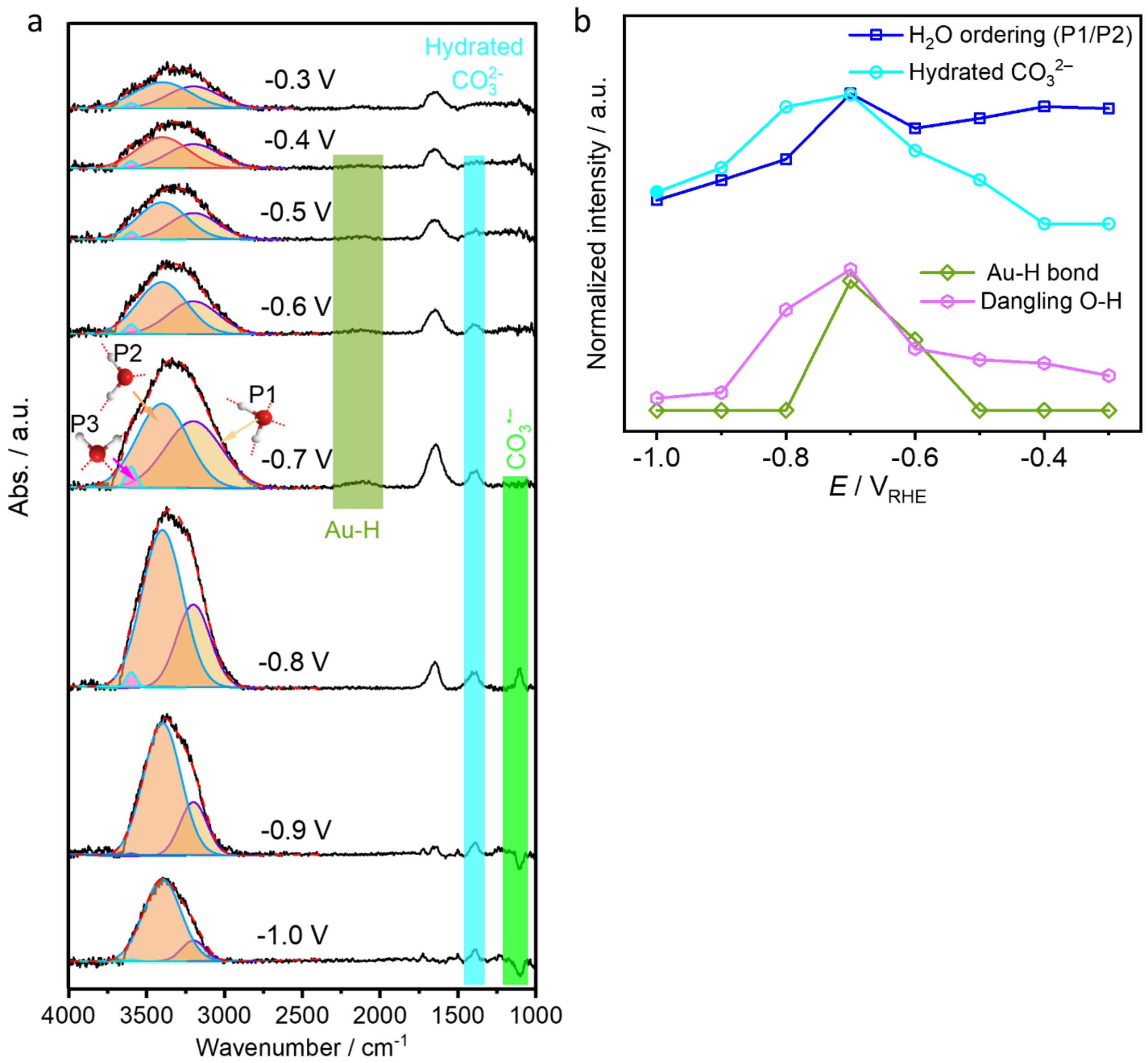

Supplementary Figure 19: (a) *In situ* ATR-SEIRA spectra of interfacial H_2O with p-polarization for polycrystalline Au electrode in Ar-saturated 0.1 M $KHCO_3$. Gaussian fits of three O-H stretching modes shown as yellow (Peak 1), orange (Peak 2) and purple (Peak 3). (b) Normalized band intensity extracted from (a).

9. Role of $CO_3^{\bullet-}$ in HER and HCHO formation

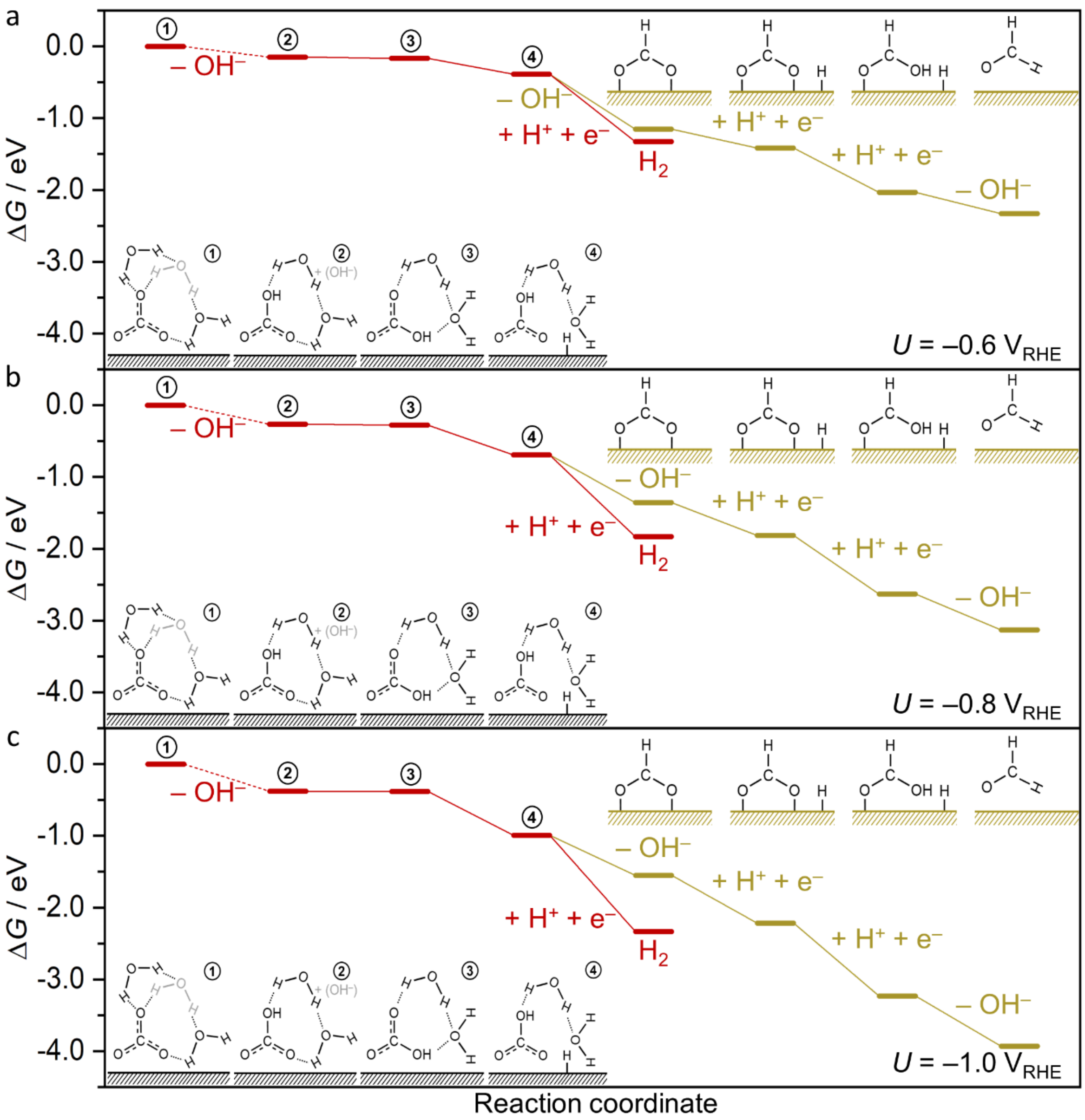

Supplementary Figure 20: Gibbs energy profiles of $CO_3^{\bullet-}$ reduction (yellow) and HER (red) on Au (100) at different potential –0.6 V_{RHE} (a), –0.8 V_{RHE} (b), and –1.0 V_{RHE} (c).

References

1 Li, C.-Y. *et al.* In situ probing electrified interfacial water structures at atomically flat surfaces. *Nature Materials* **18**, 697-701 (2019).
2 Tong, Y., Lapointe, F., Thämer, M., Wolf, M. & Campen, R. K. Hydrophobic water probed experimentally at the gold electrode/aqueous interface. *Angewandte Chemie International Edition* **56**, 4211-4214 (2017).
3 Alfarano, S. R. *et al.* Stripping away ion hydration shells in electrical double-layer formation: Water networks matter. *Proceedings of the National Academy of Sciences* **118**, e2108568118 (2021).
4 Velasco-Velez, J.-J. *et al.* The structure of interfacial water on gold electrodes studied by x-ray absorption spectroscopy. *Science* **346**, 831-834 (2014).
5 Li, J.-F. *et al.* SERS and DFT study of water on metal cathodes of silver, gold and platinum nanoparticles. *Physical Chemistry Chemical Physics* **12**, 2493-2502 (2010).
6 Nihonyanagi, S. *et al.* Potential-dependent structure of the interfacial water on the gold electrode. *Surface Science* **573**, 11-16 (2004).
7 Ataka, K.-i., Yotsuyanagi, T. & Osawa, M. Potential-dependent reorientation of water molecules at an electrode/electrolyte interface studied by surface-enhanced infrared absorption spectroscopy. *The Journal of Physical Chemistry* **100**, 10664-10672 (1996).
8 Ataka, K. I. & Osawa, M. In situ infrared study of water-sulfate coadsorption on gold(111) in sulfuric acid solutions. *Langmuir* **14**, 951-959 (1998).
9 Futamata, M. In-situ ATR-IR study of water on gold electrode surface. *Surface science* **427**, 179-183 (1999).
10 Schutte, C. J. H. & Buijs, K. The infra-red spectra of K_2CO_3 and its hydrates. *Spectrochim. Acta* **17**, 921-926 (1961).
11 Andersen, F. A. *et al.* Infrared spectra of amorphous and crystalline calcium carbonate. *Acta Chem. Scand.* **45**, 1018-1024 (1991).
12 Davis, A. & Oliver, B. A vibrational-spectroscopic study of the species present in the CO_2− H_2O system. *J. Solution Chem.* **1**, 329-339 (1972).
13 Oliver, B. G. & Davis, A. R. Vibrational spectroscopic studies of aqueous alkali metal bicarbonate and carbonate solutions. *Can. J. Chem.* **51**, 698-702 (1973).
14 Moradzaman, M. & Mul, G. Infrared analysis of interfacial phenomena during electrochemical reduction of CO2 over polycrystalline copper electrodes. *ACS Catalysis* **10**, 8049-8057 (2020).
15 Zhu, S., Jiang, B., Cai, W.-B. & Shao, M. Direct observation on reaction intermediates and the role of bicarbonate anions in CO_2 electrochemical reduction reaction on Cu surfaces. *Journal of the American Chemical Society* **139**, 15664-15667 (2017).
16 Dunwell, M. *et al.* The central role of bicarbonate in the electrochemical reduction of carbon dioxide on gold. *Journal of the American Chemical Society* **139**, 3774-3783 (2017).
17 Goyal, A. & Koper, M. T. The interrelated effect of cations and electrolyte pH on the hydrogen evolution reaction on gold electrodes in alkaline media. *Angewandte Chemie International Edition* **60**, 13452-13462 (2021).
18 Tian, Z.-Q., Ren, B., Chen, Y.-X., Zou, S.-Z. & Mao, B.-W. Probing electrode/electrolyte interfacial structure in the potential region of hydrogen evolution by Raman spectroscopy. *J. Chem. Soc., Faraday Trans.* **92**, 3829-3838 (1996).
19 Peremans, A. & Tadjeddine, A. Vibrational spectroscopy of electrochemically deposited hydrogen on platinum. *Phys. Rev. Lett.* **73**, 3010 (1994).
20 Nichols, R. J. & Bewick, A. Spectroscopic identification of the adsorbed intermediate in hydrogen evolution on platinum. *Journal of Electroanalytical Chemistry and Interfacial Electrochemistry* **243**, 445-453 (1988).
21 Wuttig, A. *et al.* Tracking a common surface-bound intermediate during CO_2-to-fuels catalysis. *ACS Central Science* **2**, 522-528 (2016).
22 Nakamura, M., Kato, H. & Hoshi, N. Infrared spectroscopy of water adsorbed on M (111)(M= Pt, Pd, Rh, Au, Cu) electrodes in sulfuric acid solution. *The Journal of Physical Chemistry C* **112**, 9458-9463 (2008).

23 Nakanaga, T., Kondo, S. & Saëki, S. Infrared band intensities of formaldehyde and formaldehyde-d 2. *The Journal of Chemical Physics* **76**, 3860-3865 (1982).
24 Marcandalli, G., Villalba, M. & Koper, M. T. M. The importance of acid–base equilibria in bicarbonate electrolytes for CO_2 electrochemical reduction and CO reoxidation studied on Au(hkl) electrodes. *Langmuir* **37**, 5707-5716 (2021).
25 Zilberg, S., Mizrahi, A., Meyerstein, D. & Kornweitz, H. Carbonate and carbonate anion radicals in aqueous solutions exist as $CO_3(H_2O)_6^{2-}$ and $CO_3(H_2O)_6^{\bullet-}$ respectively: the crucial role of the inner hydration sphere of anions in explaining their properties. *Physical Chemistry Chemical Physics* **20**, 9429-9435 (2018).
26 Armstrong, D., Waltz, W. & Rauk, A. Carbonate radical anion—Thermochemistry. *Can. J. Chem.* **84**, 1614-1619 (2006).
27 Bisby, R., Johnson, S., Parker, A. & Tavender, S. Time-resolved resonance Raman spectroscopy of the carbonate radical. *J. Chem. Soc., Faraday Trans.* **94**, 2069-2072 (1998).
28 Goyal, A. & Koper, M. Understanding the role of mass transport in tuning the hydrogen evolution kinetics on gold in alkaline media. *The Journal of Chemical Physics* **155** (2021).
29 Zhang, B. A., Ozel, T., Elias, J. S., Costentin, C. & Nocera, D. G. Interplay of Homogeneous Reactions, Mass Transport, and Kinetics in Determining Selectivity of the Reduction of CO_2 on Gold Electrodes. *ACS Central Science* **5**, 1097-1105, doi:10.1021/acscentsci.9b00302 (2019).
30 Kunimatsu, K., Senzaki, T., Samjeské, G., Tsushima, M. & Osawa, M. Hydrogen adsorption and hydrogen evolution reaction on a polycrystalline Pt electrode studied by surface-enhanced infrared absorption spectroscopy. *Electrochimica Acta* **52**, 5715-5724 (2007).
31 Sun, S.-G., Cai, W.-B., Wan, L.-J. & Osawa, M. Infrared Absorption Enhancement for CO Adsorbed on Au Films in Perchloric Acid Solutions and Effects of Surface Structure Studied by Cyclic Voltammetry, Scanning Tunneling Microscopy, and Surface-Enhanced IR Spectroscopy. *The Journal of Physical Chemistry B* **103**, 2460-2466 (1999).
32 Marcandalli, G., Villalba, M. & Koper, M. T. The importance of acid–base equilibria in bicarbonate electrolytes for CO_2 electrochemical reduction and CO reoxidation studied on Au (hkl) electrodes. *Langmuir* **37**, 5707-5716 (2021).
33 Sheng, H. *et al.* Carbon dioxide dimer radical anion as surface intermediate of photoinduced CO_2 reduction at aqueous Cu and CdSe nanoparticle catalysts by rapid-scan FT-IR spectroscopy. *Journal of the American Chemical Society* **140**, 4363-4371 (2018).
34 Chen, S. & Chen, A. Electrochemical reduction of carbon dioxide on Au nanoparticles: an in situ FTIR study. *The Journal of Physical Chemistry C* **123**, 23898-23906 (2019).
35 Firet, N. J. & Smith, W. A. Probing the Reaction Mechanism of CO2 Electroreduction over Ag Films via Operando Infrared Spectroscopy. *ACS Catalysis* **7**, 606-612 (2017).
36 Chernyshova, I. V., Somasundaran, P. & Ponnurangam, S. On the origin of the elusive first intermediate of CO_2 electroreduction. *Proceedings of the National Academy of Sciences* **115**, E9261-E9270 (2018).
37 Shao, F. *et al.* In situ spectroelectrochemical probing of CO redox landscape on copper single-crystal surfaces. *Proceedings of the National Academy of Sciences* **119**, e2118166119 (2022).
38 Monteiro, M. C. O. *et al.* Absence of CO_2 electroreduction on copper, gold and silver electrodes without metal cations in solution. *Nature Catalysis* **4**, 654-662 (2021).
39 Medinas, D. B., Cerchiaro, G., Trindade, D. F. & Augusto, O. The carbonate radical and related oxidants derived from bicarbonate buffer. *IUBMB life* **59**, 255-262 (2007).
40 Wojnárovits, L., Tóth, T. & Takács, E. Rate constants of carbonate radical anion reactions with molecules of environmental interest in aqueous solution: A review. *Sci. Total Environ.* **717**, 137219 (2020).
41 Arnold, W. A. One electron oxidation potential as a predictor of rate constants of N-containing compounds with carbonate radical and triplet excited state organic matter. *Environmental Science: Processes & Impacts* **16**, 832-838 (2014).
42 Huang, J. & Mabury, S. A. A new method for measuring carbonate radical reactivity toward pesticides. *Environmental Toxicology and Chemistry: An International Journal* **19**, 1501-1507 (2000).
43 Augusto, O. *et al.* Nitrogen dioxide and carbonate radical anion: two emerging radicals in biology. *Free Radical Biol. Med.* **32**, 841-859 (2002).

44 Huang, J. & Mabury, S. A. Steady-state concentrations of carbonate radicals in field waters. *Environmental Toxicology and Chemistry: An International Journal* **19**, 2181-2188 (2000).
45 Pan, M., Pozun, Z. D., Yu, W.-Y., Henkelman, G. & Mullins, C. B. Structure Revealing H/D Exchange with Co-Adsorbed Hydrogen and Water on Gold. *The Journal of Physical Chemistry Letters* **3**, 1894-1899 (2012).
46 Kunimatsu, K., Senzaki, T., Tsushima, M. & Osawa, M. A combined surface-enhanced infrared and electrochemical kinetics study of hydrogen adsorption and evolution on a Pt electrode. *Chem. Phys. Lett.* **401**, 451-454 (2005).
47 Kunimatsu, K., Uchida, H., Osawa, M. & Watanabe, M. In situ infrared spectroscopic and electrochemical study of hydrogen electro-oxidation on Pt electrode in sulfuric acid. *Journal of Electroanalytical Chemistry* **587**, 299-307 (2006).
48 Chen, L. D., Urushihara, M., Chan, K. & Nørskov, J. K. Electric Field Effects in Electrochemical CO2 Reduction. *ACS Catalysis* **6**, 7133-7139 (2016).
49 Ringe, S. *et al.* Understanding cation effects in electrochemical CO_2 reduction. *Energy & Environmental Science* **12**, 3001-3014 (2019).
50 Ringe, S. *et al.* Double layer charging driven carbon dioxide adsorption limits the rate of electrochemical carbon dioxide reduction on Gold. *Nature Communications* **11**, 33 (2020).
51 Chen, Y., Li, C. W. & Kanan, M. W. Aqueous CO_2 reduction at very Low overpotential on oxide-derived Au nanoparticles. *Journal of the American Chemical Society* **134**, 19969-19972 (2012).
52 Amatore, C. & Saveant, J. M. Mechanism and kinetic characteristics of the electrochemical reduction of carbon dioxide in media of low proton availability. *Journal of the American Chemical Society* **103**, 5021-5023 (1981).
53 Manthiram, K., Beberwyck, B. J. & Alivisatos, A. P. Enhanced Electrochemical Methanation of Carbon Dioxide with a Dispersible Nanoscale Copper Catalyst. *Journal of the American Chemical Society* **136**, 13319-13325 (2014).
54 Tryk, D. A. *et al.* Recent developments in electrochemical and photoelectrochemical CO_2 reduction: involvement of the (CO2) 2•– dimer radical anion. *Appl. Organomet. Chem.* **15**, 113-120 (2001).
55 Pathak, A. K. & Maity, D. K. Distinctive IR Signature of $CO_3^{\bullet-}$ and CO_3^{2-} Hydrated Clusters: A Theoretical Study. *The Journal of Physical Chemistry A* **113**, 13443-13447 (2009).
56 Gao, J., Duan, X., O'Shea, K. & Dionysiou, D. D. Degradation and transformation of bisphenol A in UV/Sodium percarbonate: Dual role of carbonate radical anion. *Water Res.* **171**, 115394 (2020).
57 Huang, J. & Mabury, S. A. Steady-state concentrations of carbonate radicals in field waters. *Environ. Toxicol. Chem.* **19**, 2181-2188 (2000).
58 Li, P. *et al.* Hydrogen bond network connectivity in the electric double layer dominates the kinetic pH effect in hydrogen electrocatalysis on Pt. *Nature Catalysis* **5**, 900-911 (2022).
